# Rapid single-molecule characterisation of enzymes involved in nucleic-acid metabolism

**DOI:** 10.1093/nar/gkac949

**Published:** 2022-11-02

**Authors:** Stefan H Mueller, Lucy J Fitschen, Afnan Shirbini, Samir M Hamdan, Lisanne M Spenkelink, Antoine M van Oijen

**Affiliations:** Molecular Horizons and School of Chemistry and Molecular Bioscience, University of Wollongong, Wollongong, New South Wales 2522, Australia; Illawarra Health & Medical Research Institute, Wollongong, New South Wales 2522, Australia; Molecular Horizons and School of Chemistry and Molecular Bioscience, University of Wollongong, Wollongong, New South Wales 2522, Australia; Illawarra Health & Medical Research Institute, Wollongong, New South Wales 2522, Australia; Biological and Environmental Science and Engineering Division, King Abdullah University of Science and Technology (KAUST), Thuwal 23955-6900, Kingdom of Saudi Arabia; Biological and Environmental Science and Engineering Division, King Abdullah University of Science and Technology (KAUST), Thuwal 23955-6900, Kingdom of Saudi Arabia; Molecular Horizons and School of Chemistry and Molecular Bioscience, University of Wollongong, Wollongong, New South Wales 2522, Australia; Illawarra Health & Medical Research Institute, Wollongong, New South Wales 2522, Australia; Molecular Horizons and School of Chemistry and Molecular Bioscience, University of Wollongong, Wollongong, New South Wales 2522, Australia; Illawarra Health & Medical Research Institute, Wollongong, New South Wales 2522, Australia

## Abstract

The activity of enzymes is traditionally characterised through bulk-phase biochemical methods that only report on population averages. Single-molecule methods are advantageous in elucidating kinetic and population heterogeneity but are often complicated, time consuming, and lack statistical power. We present a highly-generalisable and high-throughput single-molecule assay to rapidly characterise proteins involved in DNA metabolism. The assay exclusively relies on changes in total fluorescence intensity of surface-immobilised DNA templates as a result of DNA synthesis, unwinding or digestion. Combined with an automated data-analysis pipeline, our method provides enzymatic activity data of thousands of molecules in less than an hour. We demonstrate our method by characterising three fundamentally different enzyme activities: digestion by the phage *λ* exonuclease, synthesis by the phage *Phi29* polymerase, and unwinding by the *E. coli* UvrD helicase. We observe the previously unknown activity of the UvrD helicase to remove neutravidin bound to 5′-, but not 3′-ends of biotinylated DNA.

## INTRODUCTION

Maintenance of DNA, involving replication, repair and recombination, requires many different enzymes with a range of different activities. Development of information-rich biochemical assays that report on these activities is an important step towards our understanding of their molecular mechanisms in disease pathways such as anti-microbial resistance (1) and cancer (2). Additionally, characterisation of enzymes involved in nucleic-acid metabolism plays an important role in the development of methods such as gene amplification and DNA sequencing, widely used not only in molecular biochemistry, but also forensics, diagnostics (3) and palaeontology (4). Traditionally, the activity of DNA-modifying enzymes is characterised through ensemble-averaging biochemical methods, such as gel electrophoresis and fluorimetry. These methods have the drawback of averaging over large ensembles of molecules and, therefore, provide no access to information on subpopulations, dynamic molecular mechanisms and intermediate states. However, knowledge of these properties is often crucial to a full understanding of the molecular processes underlying DNA metabolism and the enzymes involved.

To describe such properties, researchers have developed techniques to observe single molecules in real time. These methods often rely on imaging fluorescent tags or manipulating molecules using optical tweezers (5). In recent years, these techniques have revealed unexpected dynamics (6–9) and quantitatively characterised interactions on the molecular scale (10,11). While these techniques have yielded new insights into molecular properties of enzymes and protein dynamics, a major disadvantage of single-molecule approaches is the time-consuming and complex nature of the experiments and data analysis needed to acquire statistically significant data. Because of these challenges, single-molecule studies are difficult to reproduce by other researchers and the statistical power of many studies is comparatively small. Here, we describe a single-molecule assay that can be used to characterise any enzyme that catalyses the conversion between double-stranded DNA (dsDNA) and single-stranded DNA (ssDNA). The assay provides kinetic information on large numbers of molecules in one experiment and is simple to implement relative to existing single-molecule experiments. By using fluorescent probes that selectively stain ssDNA or dsDNA and by monitoring fluorescence-intensity changes of surface-immobilised, randomly-coiled DNA templates, we can visualise the conversion of dsDNA to ssDNA in real time for hundreds of molecules simultaneously. In contrast to previously described methods (12) our assay is easy to modify for a range of different enzymes without major changes to our analysis pipeline.

**Figure 1.**
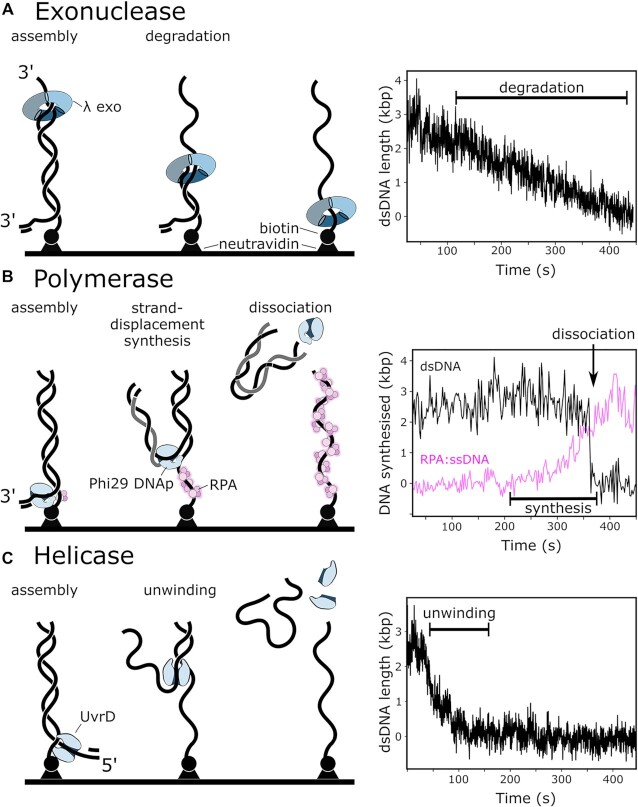
Schematic representation of the assay and example data. (**A**) Trimeric exonuclease lambda loads on free 3′ ends and converts dsDNA to ssDNA (left). This digestion leads to a decrease in measured DNA-stain intensity (right). (**B**) Phi29 DNAp mediated strand-displacement synthesis (left). Phi29 DNAp binds the primed 3′ end of the template. In the presence of dNTPs the template strand becomes single stranded, as the newly-synthesised daughter strand is displaced and eventually dissociates from the surface and therefore becomes invisible in TIRF-microscopy. This displacement leads to the instantaneous drop in DNA-stain intensity (right; black line). In parallel, we use fluorescently-labelled RPA to visualise the increasing amount of exposed ssDNA (magenta line). (**C**) UvrD helicase assembles at the available 5′ end (left). In the presence of ATP dsDNA is converted to ssDNA, leading to a decrease in DNA-stain intensity (right).

As a proof of principle, we characterise three enzymes with different functions. We visualise exonucleolytic degradation of the DNA template catalysed by phage λ exonuclease (λ exo) (Figure 1A), strand-displacement synthesis by the phage Phi29 DNA polymerase (Phi29 DNAp) (Figure 1B), and unwinding of DNA by the *Escherichia coli* UvrD helicase (Figure 1C). We report rate constants and distributions, determined by characterising the metabolism of thousands of individual DNA molecules for each of the three enzymes. The statistical power of our study greatly exceeds that of previous single-molecule studies of these enzymes (13–16), yet our assay is comparatively easy to implement on any commercially available or home-built TIRF-microscope and due to a highly automated data-analysis pipeline, less time-consuming (See Supplementary Figure S1 for details) than previously described methods. Using this assay we observed the removal of neutravidin from biotinylated 5′-, but not 3′-DNA ends by the UvrD helicase — an activity that was previously unknown.

## MATERIALS AND METHODS

### DNA template construction

As a starting material we used the 4 kbf plasmid, a plasmid 4 kb in length and derived from pUC19, previously developed by Dr Jacob Lewis. The plasmid was simultaneously digested with restriction endonucleases BsaI and BstXI (NEB). The resulting 2.6-kb fragment was separated from the 1.4-kb fragment and uncut plasmid by agarose gel purification (Promega Gel Wizzard Kit). A set of oligonucleotides that form a biotinylated and primed fork was ligated to one end of the fragment and the final product purified on a Sepharose-4B column (see Supplementary Figure S2) as previously described (17). The final product was stored at 4°C. Full plasmid map and oligonucleotide sequences are described in the Supplementary Data section (see Supplementary Tables S1 and S2).

### Preparation of microfluidic flow cells

Flow chambers for microscopy were prepared as described before (7,18,19). Briefly, cover slips (24 × 24 mm, Marienfeld) were functionalised with biotin-PEG (Laysan Bio). A polydimethylsiloxane (PDMS) block was made using soft-lithography methods and placed on top of the cover slips, creating a 1-mm wide and 0.1-mm high channel with a volume of 1 μl. Two stretches of polyethylene tubing (PE-60: 0.76-mm inlet diameter and 1.22-mm outer diameter, Walker Scientific) were inserted into the PDMS block at the entrance and exit of the channel to allow for buffer flow. Before the start of experiments, the flow channel was incubated with blocking buffer (50 mM Tris–HCl pH 7.6, 50 mM Potassium Chloride, 2% (v/v) Tween-20) to minimise nonspecific binding of DNA or proteins to the cover-slip surface. A syringe pump (Adelab Scientific) was used to introduce solutions to the flow cell.

### TIRF microscopy

The flow-cell device was mounted on an inverted total-internal reflection fluorescence (TIRF) microscope (Nikon Eclipse Ti-E), with an electrically heated stage (31°C unless specified; Okolab) and a 100x TIRF objective (NA = 1.49, oil, Nikon). Samples were illuminated using a 514-nm laser (Coherent, Sapphire 514–150 CW) at 1.6 mW cm^−2^ and a 647-nm laser (Coherent, Obis 647–100 CW) at 5.2 mW cm^−2^. The fluorescence signals were captured with an EMCCD camera (Hamamatsu C9100-13) through a dual-band emission filter (TRF59907-EM, Chroma). For all measurements involving labelled RPA, samples were visualised at a frame rate of 0.5 frames per second with an exposure time of 400 ms. For λ exo and UvrD reactions samples were visualised at a frame rate of 5 frames per second with an exposure time of 200 ms, unless otherwise specified.

### λ exonuclease reactions

Firstly, 140 μl of 20 pM forked DNA template (Substrate 1) in Replication Buffer (25 mM Tris–HCl, pH 7.6, 10 mM magnesium acetate, 50 mM potassium glutamate, 40 μg/ml BSA, 0.1 mM EDTA, 5 mM dithiothreitol, and 0.0025% (v/v) Tween-20) in the presence of 150 nM SYTOX orange (Life Technologies) was loaded into the flowcell at a rate of 70 μl/min. After 1 min or after a density of 0.3–0.7 molecules/μm^2^ on the surface was reached, 140 μl of Replication Buffer with 150 nM SYTOX orange and 20 nM RPA was loaded at a flow rate of 70 μl/min. Finally, 10 units of λ exonuclease (NEB) diluted in 80 μl sof Replication Buffer supplemented with 150 nM SYTOX orange and 20 nM RPA were loaded into the flowcell at 70 μl/min.

### Preparation of AF647-RPA

Purified RPA was a generous gift from Dr Michael O’Donnell, fluorescent labelling of RPA was performed as previously described (7). Briefly, 45 μM RPA in 550 μl of RPA labeling buffer (50 mM Tris–HCl pH 7.6, 3 mM dithiothreitol, 1 mM EDTA, 200 mM NaCl, 10% (v/v) glycerol) was incubated at 5-fold molar excess of Alexa Fluor 647 (Invitrogen) for 2 h at 23°C with gentle rotation. Immediately following the coupling, excess dye was removed by gel filtration at 1 ml/min through a column (1.5 × 10 cm) of Sephadex G-25 (GE Healthcare), equilibrated in gel filtration buffer (50 mM Tris–HCl pH 7.6, 3 mM dithiothreitol, 1 mM EDTA, 200 mM NaCl, 20% (v/v) glycerol). Labelled AF647–RPA was frozen in liquid N_2_ and stored as single use aliquots at –80°C. The degree of labeling was measured to be 1 fluorophore per RPA trimer by UV/vis spectrophotometry.

### Strand-displacement reactions

First, the forked DNA template (20 pM in Replication Buffer) was loaded into the channel at a rate of 70 μl/min in the presence of 150 nM SYTOX orange, allowing for direct visualisation. After 1 minute or after a density of 0.3–0.7 molecules/μm^2^ on the surface was reached, 80 μl of fluorescently labelled RPA (AF647-RPA, 20 nM in Replication Buffer supplemented with 150 nM SYTOX orange) was loaded at a rate of 70 μl/min. Before the reaction was initiated, initial fluorescence intensities were recorded to determine the base line of RPA intensity at the fork. Next, 5 units of Phi29 DNAp (NEB) was loaded in the presence of 20 nM RPA and the specified concentration of dNTPs.

### Detection of events and post-synchronisation

Data analysis was carried out using Fiji (20) and Python. The raw data was first corrected for a non-uniform excitation-beam profile and mechanical drift of the microscope stage during the measurement (see Supplementary Figures S3 and S4). Next, all fluorescent spots corresponding to DNA templates bound to the surface were detected using a threshold approach (see Supplementary Figure S5) and the intensity of the DNA, and if present RPA, was measured over time. Next, all trajectories were fitted with the following piecewise-linear function with three segments:

(1) \begin{eqnarray*}f \left( x \right) = \left\{ \begin{array}{@{}*{1}{c}@{}} {ma + t, \, x < a}\\ {mx + t, \, a \le x \le b}\\ {mb + t, \, x > b} \end{array}\right. \end{eqnarray*}

The parameter *a* denotes the time when enzymatic activity begins and the slope changes from 0 to a constant value *m*. The parameter *b* denotes the time when the whole substrate was processed and the slope becomes 0 again. The intensity during the first segment (*x < a*) corresponds to 2620 bp (or 0 bp in RPA trajectories), the last segment corresponds to 0 bp (or 2620 bp for RPA trajectories). The calibrated value *I*_cal_ is then given by:

(2) \begin{eqnarray*}{I}_{cal} = \frac{{I - \left( {mb + t} \right)}}{{\left( {ma + t} \right) - \left( {mb + t} \right)}}\ \end{eqnarray*}

where *I* denotes the raw fluorescence intensity. Again, for RPA trajectories increasing in intensity *a* has to be substituted with *b* and vice versa. Incomplete reactions, as determined by either negative parameters *a* or *b* or parameters *a* or *b* greater than the number of frames in a movie, were discarded. For Phi29 DNAp trajectories completion of replication was additionally confirmed by the dissociation of the newly synthesised double-stranded DNA from the now single-stranded template that remains bound to the cover slip surface. This dissociation results in a sudden drop of intensity, detected by applying a regression tree algorithm (21,22). Trajectories considered for further analysis showed a coefficient of determination higher than 0.7. By defining the time of DNA dissociation for Phi29 trajectories or the parameter *b* for *λ* exonuclease (see Supplementary Figure S6) as time point zero, we synchronised trajectories at a well-defined time point corresponding to the end of the reaction (post-reaction synchronisation). Finally, we calculated the mean intensity in both channels at every time point, both before and after time point zero. Rate distributions for λ exonuclease and UvrD were calculated by fitting the trajectories again to Equation (1), the absolute of fit parameter *m* then determines the rate.

### Determination of DNA synthesis rate by Phi29 DNAp

To determine the rate of DNA synthesis by Phi29 DNAp the post-synchronised trajectories were fit to to single exponential functions of the form:

(3) \begin{eqnarray*}f\left( x \right) = {e}^{k\left( {x - {x}_0} \right)} \end{eqnarray*}

where *k* denotes the rate constant in s^−1^, and *x*_0_ shifts the function to negative time values. Values for *x* > 0, i.e. after dissociation of the daughter strand where ignored for fits, since RPA fluorescence signal saturates and is no longer described by a single-exponential function. To calculate a rate in bp·s^−1^ we multiplied the values with the length of the used substrate (2620 bp). To determine a Michaelis-Menten constant we plotted the obtained rates over the used dNTP concentration and fitted the data to the Michaelis–Menten equation:

(4) \begin{eqnarray*}v\left( {\left[ {{\rm dNTPs}} \right]} \right) = \frac{{{v}_{{\rm max}}\left[ {{\rm dNTPs}} \right]}}{{{K}_{\rm M} + \left[ {{\rm dNTPs}} \right]}} \end{eqnarray*}

where *v* denotes the synthesis rate as a function of dNTP concentration ([dNTPs]), *v*_max_ denotes the synthesis rate in saturating dNTP concentrations and *K*_M_ the Michaelis–Menten constant, the dNTP concentration at which the rate is half the saturation rate. Note that we assume an identical affinity for each of the four dNTPs.

### Determination of RPA association constant

We treated RPA binding to ssDNA as a first-order reaction. Such reactions are described by a differential equation of the form:

(5) \begin{eqnarray*}\frac{{{\rm d}\left[ {{\rm ssDNA}_{{\rm free}}} \right]}}{{{\rm d}x}} = - {k}_{{\rm on}}\left[ {{\rm ssDNA}_{{\rm free}}} \right]\left( x \right)\end{eqnarray*}

where [ssDNA_free_] is the amount of free ssDNA that allows RPA binding and *k*_on_ denotes the molecular rate association constant of RPA to ssDNA. By integration one finds:

(6) \begin{eqnarray*}\left[ {{\rm ssDNA}_{{\rm free}}} \right] \left( x \right) = {\left[ {{\rm ssDNA}_{{\rm free}}} \right]}_{x = 0} {{\rm e}}^{ - {k}_{{\rm on}}x}\end{eqnarray*}

where [ssDNA_free_]_*x* = 0_ is the amount of initially available ssDNA. The total amount of ssDNA ([ssDNA_total_]), free or bound cannot exceed the length of the template. It follows:

(7) \begin{eqnarray*} && [{\rm ssDNA}_{{\rm total}}] = \left[ {\rm ssDNA}_{\rm free} \right] \left( x \right) \nonumber \\ && \quad + \, \left[ {{\rm ssDN{A}}_{{\rm RPA}}} \right]\left( x \right) = 2620\ {\rm nt} \end{eqnarray*}

For the amount of RPA-bound ssDNA at any given time then follows:

(8) \begin{eqnarray*}\left[ {{\rm ssDN{A}}_{{\rm RPA}}} \right] \left( t \right) = 2620 - {\left[ {{\rm ssDN{A}}_{{\rm free}}} \right]}_{x = 0}{{\rm e}}^{ - {k}_{{\rm on}}x}\end{eqnarray*}

For fitting we introduced a time-offset *x*_0_ to account for the negative time values, by substituting *x* with (*x – x*_0_). This first order kintic only describes the reaction as the signal reaches saturation. We excluded any values for $x < \ - 20\ {\rm s}$ for the purpose of fitting.

### UvrD cloning, expression and purification

The sequence-optimised UvrD gene tagged with 8xHis-tag at the N-terminus was cloned into pE-SUMO expression vector (Lifesensors Inc.) using Gibson reaction. The plasmid was then transformed by heat shock into BL21(DE3)pLysS *E. coli* competent cells. Four liters of 2YT media with a final concentration of 50 μg/ml kanamycin were inoculated with 20 ml of an overnight culture of the transformed *E. coli* cells and incubated with shaking at 30°C till reaching OD_600_ ∼0.6. The overexpression of UvrD was induced by 0.5 mM Isopropyl β-d-1-thiogalactopyranoside (IPTG) concentration after which the culture was incubated with shaking at 27°C for the period of 4 h. The cells were then isolated by centrifugation at 5500 × *g* for 10 min. The resulting pellet was resuspended into Lysis buffer (50 mM Tris–HCl pH 8, 500 mM NaCl, 40 mM Imidazole, 5 mM β-mercaptoethanol (BME), 5% glycerol and EDTA-free protease inhibitor cocktail tablet per 50 ml buffer). Subsequently, the cells were lysed by adding lysozyme to the final concentration of 2 mg/ml and kept at 4°C for 30 min followed by sonication. The crude lysate was then clarified by centrifugation at 95 000 × *g*, for 1 h at 4 °C. The supernatant was directly loaded onto HiTrap HP 5 ml affinity column (GE Healthcare) pre-equilibrated with Buffer A (50 mM Tris–HCl pH 8, 500 mM NaCl, 40 mM Imidazole, 5 mM BME and 5% glycerol). The column was washed with 50 ml of Buffer A afterwards the bound protein was eluted using linear gradient against Buffer B (50 mM Tris–HCl pH 8, 500 mM NaCl, 750 mM Imidazole, 5 mM BME and 5% glycerol). The eluted fractions containing His_8_-SUMO-UvrD were pooled and incubated with SUMO protease for 16 h at 4°C to cleave the SUMO-tag and release native UvrD. After the digestion with SUMO protease, the solution was then loaded onto HiTrap HP 5 ml affinity column (GE Healthcare) pre-equilibrated with Buffer A. The flow-through fractions containing native UvrD were pooled and dialysed against storage buffer (50 mM Tris–HCl pH 8, 500 mM NaCl, 5 mM BME and 50% glycerol). The protein solution was further concentrated, flash frozen in liquid nitrogen, and stored at –80°C. The protein concentration was calculated by measuring the absorbance at 280 nm and using the theoretical molecular extension coefficient estimated from the amino-acid sequence of UvrD (105 770 M^−1^ cm^−1^).

### UvrD helicase reactions

For neutravidin-displacement Substrate 1 or 2 was loaded into the flowcell (20 pM in Replicaiton Buffer supplemented with 150nM SYTOX orange) at a rate of 70 μl/min. For unwinding reactions Substrate 3 was loaded under the same conditions. After 1 minute or after a density of 0.3–0.7 molecules/μm^2^ on the surface was reached, the flowcell was washed with 140 μl of replication buffer at a rate of 70 μl/min. To remove excess DNA molecules from solution the flowcell was washed with 140 μl of replication buffer, for reactions on Substrate 1 or Substrate 2, supplemented with 100 nM UvrD. Finally, the reaction was initiated by loading the specified concentration of ATP in Replication buffer supplemented with 150 nM SYTOX orange. Unwinding by UvrD was visualised with a constant buffer flow of 10 μl/min.

### Determination of reaction intermediates in displacement of neutravidin by UvrD

To determine the number of intermediate steps in the reactions, we fit the data to gamma-distributions of the form:

(9) \begin{eqnarray*}f \left( {x,n,k} \right) = \frac{{{x}^{n - 1}{e}^{ - k\left( {x - {x}_0} \right)}{k}^n}}{{{\mathrm{\Gamma }}\left( n \right)}} \end{eqnarray*}

The parameter *n* denotes the number of reaction intermediates with rate constant *k*. *Γ*(*n*) = (*n* − 1)! is the Gamma function, the value *x*_0_ shifts the distribution along the x-axis and was fixed to 20 s for fitting, to account for the time when ATP reaches the flowcell and the reaction starts. Fitting was performed using the maxium likelihood estimation from the python package SciPy (23).

## RESULTS

### Single-molecule characterisation of λ exonuclease activity

Our assay uses easily-constructed 2.6-kb linear dsDNA templates. These templates have a biotin on one end to allow for attachment to the surface of a microfluidic flow chamber through biotin-neutravidin binding. After surface immobolisation of the templates, we stain the dsDNA by introducing the DNA-intercalating dye SYTOX Orange into the flow cell. Finally, we initiate the enzymatic reaction by adding the enzyme and required cofactors. The activity of any enzyme that alters the amount of dsDNA can be monitored by measuring SYTOX Orange fluorescence intensity, assuming that the presence of a DNA intercalating dye itself does not drastically change the enzymatic activity.

**Figure 2.**
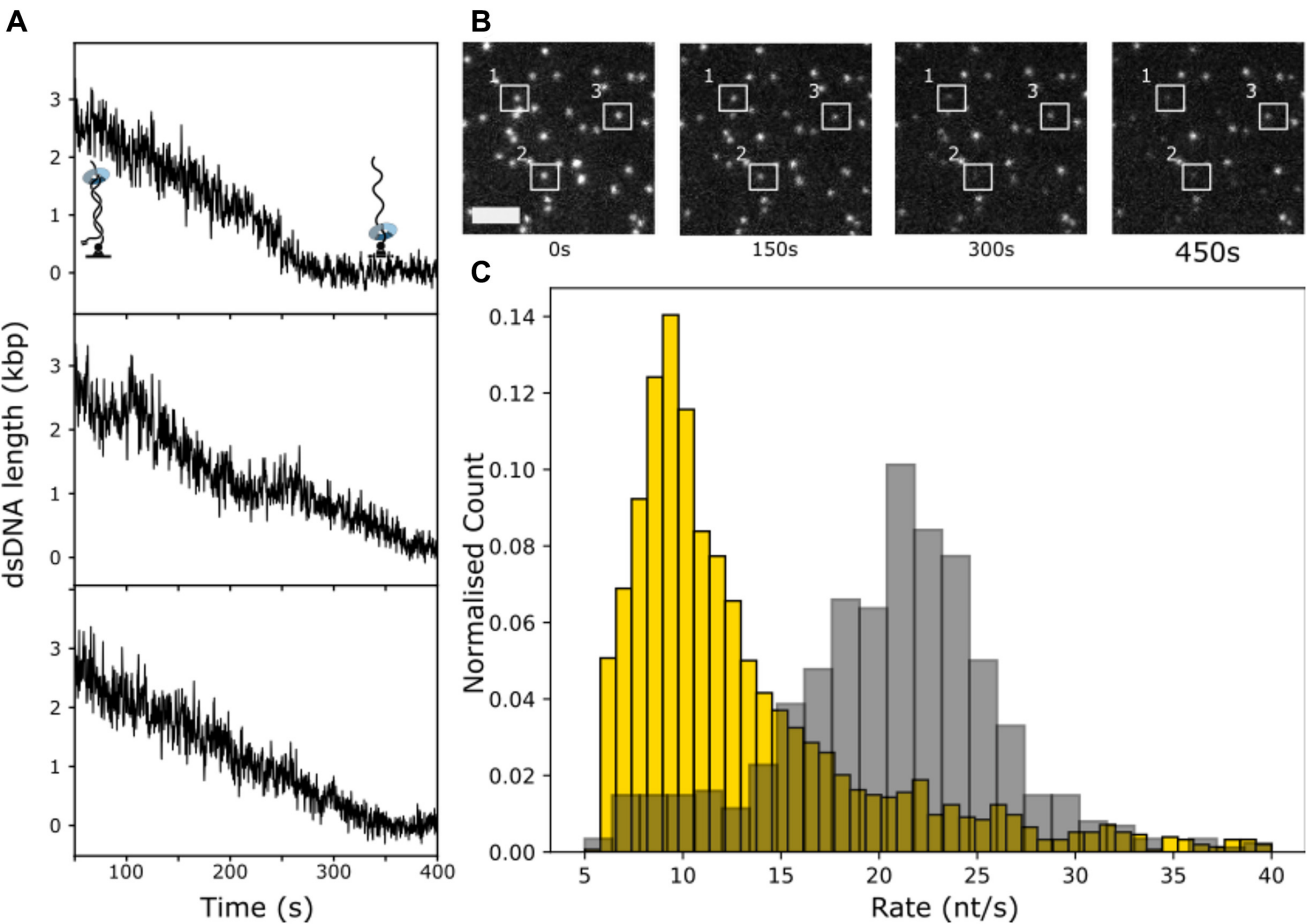
λ Exonuclease. (**A**) Single-molecule trajectories of DNA degradation by λ exonuclease. (**B**) Still frames from a recorded movie with the white rectangles numbered 1–3 as a visual guide and marking identical molecules over time, showing their decrease in DNA-stain intensity. Scale bar: 5 μm. (**C**) Rate distributions of DNA degradation by λ exonuclease at 25°C (yellow, *n* = 1957 molecules) and 35°C (grey, *n* = 648 molecules) frim three independent experiments each. See Supplementary Figure S7 for distributions of individual experiments.

The trimeric λ exo catalyses the removal of nucleotides from linear or nicked dsDNA in the 5′ to 3′ direction. During degradation of DNA, the enzyme encircles both strands (24,25). On our template, λ exo loads at the free, non-tethered end and subsequently converts the dsDNA to ssDNA by digesting nucleotides from the 5′ end (Figure 1A). As dsDNA is converted to ssDNA staining by SYTOX Orange becomes much weaker. We can therefore monitor the digestion of dsDNA in real-time by integrating the DNA-stain intensity for each individual molecule over time (see Figure 2A, B). Over a total of six experiments we record trajectories of over 2500 individual molecules. Our method is sensitive to complex stochastic kinetics of individual molecules, such as pausing (see Figure 2A, middle trajectory). However, the vast majority of trajectories shows very uniform and linear behaviour. We use piecewise linear fits to determine the digestion rate for individual molecules. We find rate distributions with means of 10.9 ± 7.1 and 21.4 ± 6.2 (mean ± standard deviation (STD)) for reactions at 25 or 35°C, respectively (see Figure 2C), consistent with previously measured values (13,26,27). The large standard deviation of the observed distributions highlights the presence of intermolecular disorder. Such effects have previously been studied by single-molecule techniques, but typically with much smaller sample sizes (13,19,28).

### Single-molecule characterisation of strand-displacement synthesis by the Phi29 DNA polymerase and ssDNA-binding properties of RPA

**Figure 3.**
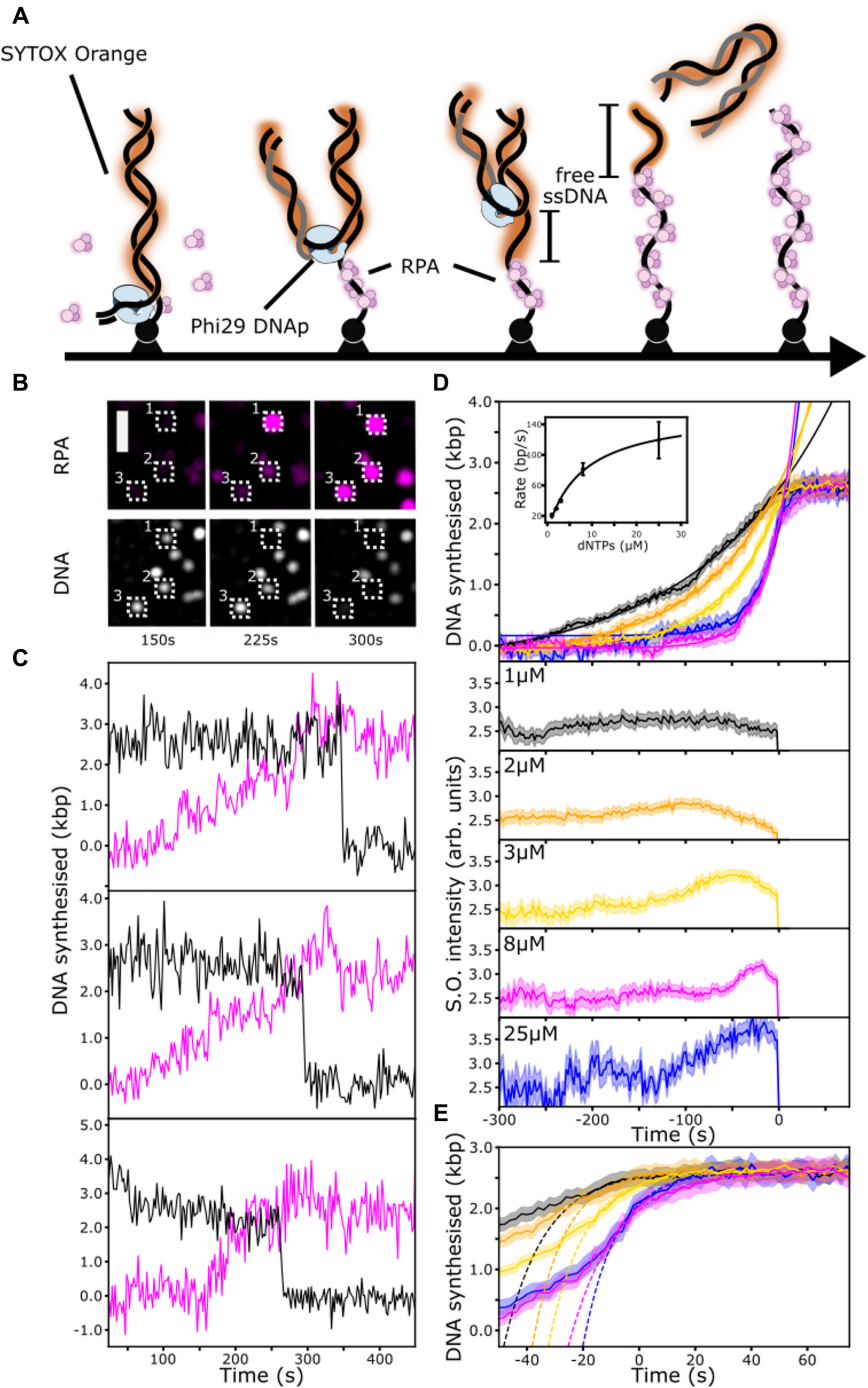
Single-molecule characterisation of Phi29 DNAp. (**A**) Schematic representation of the assay. First, Phi29 DNAp assembles on the primed DNA end. As ssDNA is created, free ssDNA accumulates before it is bound by RPA. Eventually the daughter strand dissociates, leaving behind ssDNA bound by RPA. (**B**) Montage of a TIRFM movie of strand-displacement synthesis over 300 s. The RPA signal (magenta, top) increases in intensity, while the dsDNA signal (white, bottom) stays approximately constant until it finally disappears. Scale bar: 2 μm. (**C**) Three individual single-molecule trajectories of strand-displacement synthesis. (dsDNA signal: black, ssDNA/RPA signal: magenta). (**D**) Post-synchronised average trajectories at dNTP concentrations (each) of 1 μM (black, n = 101 molecules), 2 μM (orange, n = 153 molecules), 3 μM (yellow, n = 134 molecules), 8 μM (magenta, n = 127 molecules) and 25 μM (blue, *n* = 58 molecules). The shaded area around the lines depicts the standard error of the mean (SEM). The top graph shows the RPA signal, normalised to the length of the template DNA (2620 bp). Solid smooth lines are exponential fits (see methods) of the data, restricted to time values smaller than -10 seconds. Rate constants were obtained by multiplying with the length of the template DNA. The average rate in nt·s^− 1^ was determined from three independent experiments and is shown in the inset. The error bars show the standard error of the mean. The solid line in the inset is a fit to the Michaelis-Menten equation (see methods) yielding: *K*_M_= 8 ± 3 μM, *v*_max_= 158 ± 25 bp·s^−1^. The bottom five graphs show the synchronised DNA-stain signals over time. (**E**) The increase in RPA signal prior to dissociation of the daughter strand. The dashed lines are fits to first-order rate equations (see methods). The extracted association constant from a total of 15 datasets is 11.1 ± 0.9 nt·nM^−1^·s^−1^ (mean ± SEM).

During strand-displacement synthesis by Phi29 DNAp, the net amount of dsDNA stays constant until the template strand is fully replicated and the daughter strand dissociates from the template (see Figure 3A). This dissociation is visible as a sudden drop in DNA-stain intensity. To visualise the kinetics of DNA synthesis, we additionally introduce fluorescently-labelled *S. cerevisiae* replication protein A (RPA), a single-stranded DNA-binding protein with very high affinity for ssDNA. Furthermore, while free RPA is present in solution, bound RPA exchanges rapidly (29,30). This effect mitigates photobleaching and makes RPA a good marker for ssDNA (see Supplementary Figure S8). For every synthesised nucleotide on the daughter strand, one nucleotide of ssDNA is left behind on the surface-tethered strand. Therefore, the change in RPA signal over time corresponds to the replication rate by Phi29 DNAp, knowing that RPA binds ssDNA faster than new dsDNA is synthesised (31). Figure 3C shows data from our experiments. Surprisingly, most single-molecule trajectories seemed to exhibit a non-linear dependence between RPA signal and time. However, for individual traces this behaviour is difficult to distinguish from statistical noise and pausing kinetics. To increase our signal-to-noise ratio we synchronised all trajectories to the time of dissociation of the daughter strand, which is an event that is easy to identify. Subsequent averaging across many single-molecule traces yields a synchronised average trajectory that does not suffer from the same caveats as ensemble-averaging methods and contains information on the underlying kinetics (32,33). Indeed, the post-synchronised average trajectory clearly exhibits non-linear behaviour (see Figure 3D). As a control, to prove that this is not an artefact of our analysis, we synchronised trajectories from the previous experiments on λ exonuclease (see Supplementary Figure S2). Unlike for Phi29 DNAp, the synchronised trajectory of λ exonuclease is linear. The observed non-linear Phi29 DNAp dynamics become more obvious for higher dNTP concentrations, i.e. at higher replication rates (see Figure 3D). This observation suggests that our initial assumption, that RPA binding kinetics are faster than DNA synthesis, is not generally true. Our data is well described by single-exponential functions, with a *K_M_* of Phi29 DNAp for dNTPs of 8 ± 3 μM (mean ± SEM) and a maximum synthesis rate of 160 ± 25 bp·s^−1^ (mean ± SEM, see Figure 3D). Our *K*_M_ value is about four times lower than previously reported values and our *v*_max_ value is consistent with previous measurements (34). The fact that we find lower *K*_M_ values than expected is in agreement with the hypothesis that for high dNTP concentrations, the *observed* kinetics are limited by RPA binding. This is also confirmed by the fact that our estimated *v*_max_ is in good agreement with the literature, since the maximum increase in RPA signal is still limited by strand-displacement synthesis by Phi29 DNAp.

To gain information on RPA binding kinetics within our system we examine the increase in RPA fluorescence signal immediately before and after the dissociation of the daughter strand (see Figure 3E). At the time of dissociation, Phi29 has converted the enitre surface-bound DNA template to single-stranded DNA, yet the signal from fluorescent RPA has not yet reached saturation (see Figure 3D, E). Therefore, in this regime RPA binding is no longer limited by the conversion of double-stranded to single-stranded DNA by Phi29 DNAp activity. The observed data are well described by first-order binding kinetics and yield a bimolecular association rate constant *k*_on_ of 11.1 ± 0.9 nt·nM^−1^·s^−1^ (mean ± SEM), consistent with previously reported values (31,35). A first-order kinetic model for RPA binding implies that the speed of binding at any given time depends on the number of binding partners available (see Materials and Methods). We therefore hypothesise that in the very beginning of the reaction almost no free ssDNA is present, and RPA binding is therefore slow. As replication proceeds ssDNA is generated. As more binding sites become available, RPA binding becomes faster, the amount of ssDNA decreases again, and binding slowly converges to saturation as the daughter strand dissociates (see Figure 3A). This picture implies a fluctuation of the amount of free ssDNA, not bound by RPA but very weakly stained by SYTOX orange. Since ssDNA staining is much less efficient than dsDNA, such a minor increase is not visible in the individual single-molecule trajectories. However, the post-synchronised trajectories of the SYTOX orange signal (see Figure 3D) indeed show a clear fluctuation in intensity. Our data indicates that RPA binding is stimulated by the amount of free ssDNA, and that RPA displaces SYTOX orange from ssDNA.

### Characterisation of the *E. coli* UvrD helicase

Next, we sought to test if our assay is suitable for the study of helicases. Helicases are one of the biggest families of proteins, present in all domains of life. As an example we characterise the *E. coli* UvrD helicase, a member of the SF1 family of helicases. Apart from unwinding DNA in 3′ to 5′ direction in its dimeric form, it is also involved in methyl-directed mismatch repair and acts as an anti-recombinase by removing recA filaments from ssDNA (36–38). The monomeric form of UvrD processively translocates on ssDNA (16).

**Figure 4.**
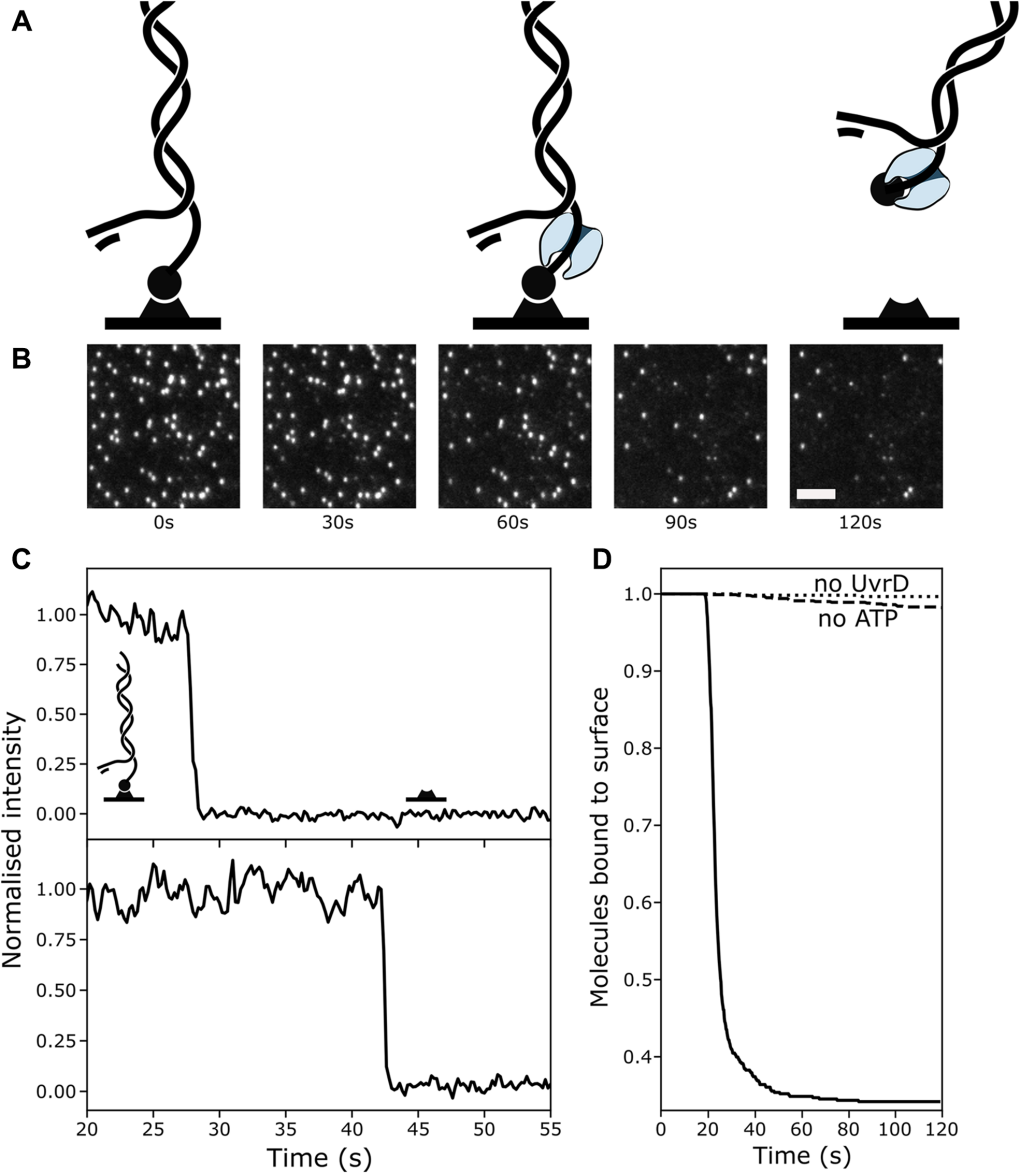
UvrD disrupts biotin-neutravidin interaction (**A**) Schematic representation of UvrD assembly next to the tethered 5 end and subsequent dissociation of the DNA substrate. (**B**) Montage of a recorded movie. The bright spots correspond to individual DNA molecules disappearing over time. (**C**) Integrated fluorescence intensities over time from the movie shown in (A). The drop in intensity corresponds to the removal of the neutravidin from the biotinylated DNA end. (**D**) Fraction of DNA substrates still bound to the surface over time, in presence of UvrD and ATP (solid line, n = 588 molecules), absence of UvrD or ATP respectively (dotted line, n = 1004, dashed line n = 583 molecules).

At first, we wanted to study ATP-dependent unwinding by the UvrD helicase on the previously used 2.6-kb forked template. We expected unwinding of DNA, and therefore a continuous decrease in the fluorescence intensity of SYTOX orange stained template DNA, as UvrD unwinds the substrate, potentially from both ends. Surprisingly, instead of the expected continuous decrease in intensity, we observe a discrete drop in fluorescence intensity, i.e. diffraction limited spots simply disappear (see Figure 4A). This observation indicates dissociation of the full template from the cover slide surface rather than DNA unwinding. Together with control reactions lacking either ATP or UvrD, this shows that the UvrD helicase can actively remove the neutravidin bound to the 5′-DNA end. We repeated the experiment at time resolutions of up to 30 frames per second. (See Supplementary Figure S9).

The trajectories still show discrete fluorescence drops within one frame, suggesting a dissociative process that is completed within 30 ms. UvrD loading on the free 3′-DNA end and unwinding DNA towards the surface within 30 ms would correspond to a rate of 80 000 nt·s^−1^. Since this high rate would be in stark contradiction to the literature (15,37,39,40), we conclude that UvrD is loading in close proximity to the surface and exhibits an enzymatic activity different to DNA unwinding.

**Figure 5.**
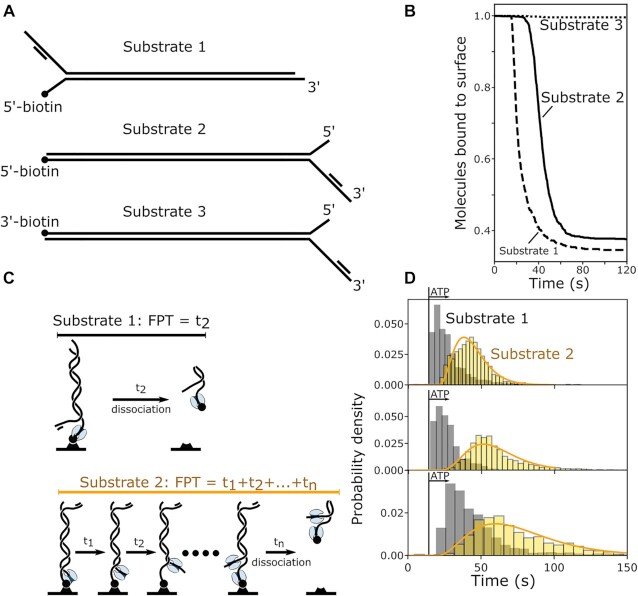
Substrate and ATP dependence of UvrD-mediated removal of neutravidin. (**A**) Schematic overview of DNA substrates. Substrate 1: 5′ biotin with ssDNA overhang, Substrate 2: 5′ biotin on blunt end, Substrate 3: 3′ biotin on blunt end. (**B**) Fraction of molecules bound to the coverslide surface over time for substrates 1–3 in presence of 50 μM ATP and 100 nM UvrD. (**C**) Principle of the First Passage Time (FPT) for a reaction without intermediate steps (top), resulting in exponentially distributed FPT distributions. For a reaction with n intermediate steps (bottom), the measured FPT equals the sum of intermediate steps. If all intermediate steps follow identical kinetics, the FPT distribution follows a gamma distribution. (**D**) FPT distributions for Substrates 1 (grey) and 2 (yellow) in presence of 100 nM UvrD and 50 μM ATP (top, Substrate 1: n = 1171 molecules, Substrate2 : n = 2534 molecules, three individual experiments), 25 μM ATP (middle, Substrate 1: n = 817 molecules, Substrate2: n = 2372 molecules, three individual experiments) and 12.5 μM ATP (bottom, Substrate 1: n = 973 molecules, Substrate2 : n = 1440 molecules, two individual experiments). The vertical bar represents the time when ATP is first present in the flowcell. Note that the first bins of the histograms are likely underrepresentative of the distributions for two reasons: First, ATP concentration within the flowcell gradually increases to the given value and second, flow is operated manually which leads to an uncertainty of the time of ATP arrival in the order of seconds. The histograms contain data from three experiments each, with slightly different ATP arrival times. Orange lines are maximum-likelyhood estimations to gamma distributions, with rate parameter k and shape parameter n (50 μM: k = 0.18 s^−1^, n = 4.2; 25 μM: k = 0.12 s^−1^, n = 4.8; 12.5 μM: k = 0.06 s^−1^, n = 3.4;). See Supplementary Figure S10 for distributions and fit parameters of individual experiments.

Next, we wanted to understand if loading on ssDNA is required for the removal of neutravidin. To do so, we made two different versions of our previous DNA substrate (henceforth referred to as Substrate 1). First, we removed the ssDNA region adjacent to the tethered 5′-end (Substrate 2) to examine if displacement of protein blocks required UvrD assembly on ssDNA. Second, we placed the biotin on the 3′-DNA end (Substrate 3), to see if this activity has the same 3′–5′ directionality as unwinding and translocation on ssDNA (see Figure 5A). DNA unwinding by UvrD was previously reported to be inefficient, if initiated from short 3′ overhangs or even blunt ends. Surprisingly, the removal of a neutravidin block is efficient, even from blunt ends (Figure 5B, solid line). However, neutravidin bound to 3′-biotinilated DNA cannot be displaced by UvrD at all (see Figure 5B, dotted line).

To gain more insight in the mechanism involved, we calculated first-passage time (FPT) distributions. FPT distributions are a powerful analysis tool, widely used to analyse and model stochastic processes, such as animal migration, the spread of COVID-19 virus particles and also helicase dynamics (41–43). The FPT *t_n_* is the time from the start (addition of ATP) to the end of a reaction (dissociation of the DNA template) for an individual molecule (see Figure 5C). The distribution of FPTs conveys information on the number and rate constants of all rate-limiting steps during the reaction (44). We preincubate Substrate 1 with UvrD and subsequently initiate the reaction by adding ATP. For Substrate 2, we observe a single-exponential FPT-distribution, a hallmark of the absence of intermediate steps (see Figure 5C grey histograms). For Substrate 2, which lacks available ssDNA for UvrD to assemble close to the 5′ end, the FPTs are well described by a gamma distribution (see Figure 5B and C, yellow histrograms). This observation indicates the presence of multiple slow reaction intermediates required to remove the neutravidin (44). Since the mean of the measured FPT distributions is much longer for Substrate 2 than for Substrate 1, we conclude that the rate-limiting steps in this case correspond to unwinding of the template from the blunt end (away from the surface), to subsequently allow for UvrD binding on the 5′ end next to the biotin. To obtain the number of reaction intermediates and corresponding rate-constants, we fit the data with gamma-distributions, as previously described (44) (see Figure 5D and Materials and Methods). Our data suggest four intermediate steps, a number that does not vary with ATP concentration. Taken together with reported step sizes of unwinding by UvrD of 3–6 nucleotides (34,45) our data indicates that unwinding of 12–24 nt is required for subsequent displacement of neutravidin.

**Figure 6.**
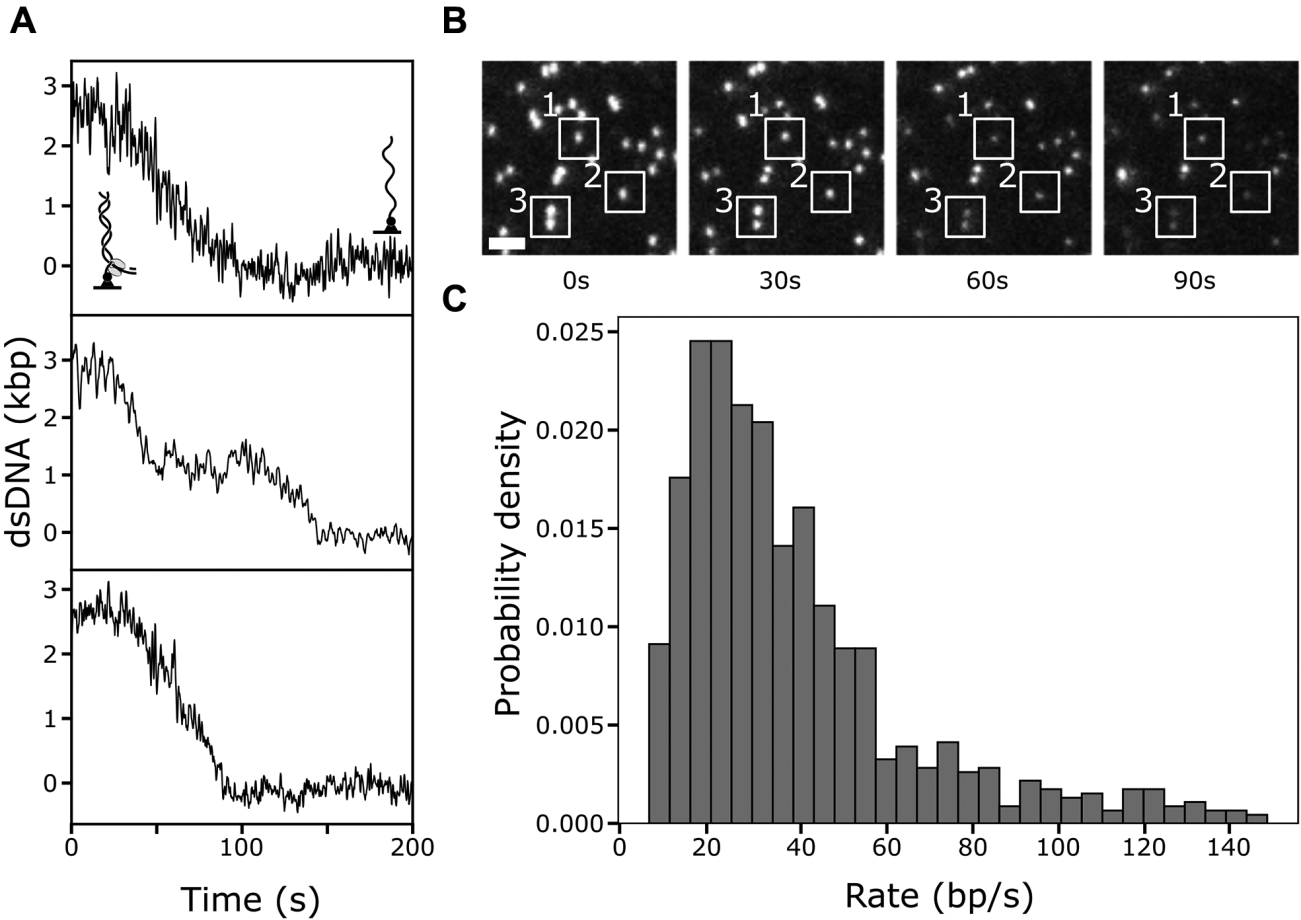
DNA unwinding by the UvrD helicase. (**A**) Single-molecule trajectories of DNA unwinding by UvrD. (**B**) Montage of TIRFM movie over 90s. Scale bar: 3 μm. The white rectangles serve as visual guide and mark individual molecules over time. (**C**) Rate distribution of DNA unwinding in the presence of 0.5 mM ATP at 25°C. n = 452 molecules.

Finally, we set out to observe DNA unwinding by UvrD. To do so, we utilise Substrate 3 (see Figure 5A). The 3′-biotin prevents disruption of the biotin-neutravidin bond, while UvrD can load on the opposite end. The 60-nt 3′-dT tail provides a substrate for UvrD dimer assembly and initiation of DNA unwinding in presence of ATP. Unwinding by UvrD results in a gradual reduction of the DNA-stain intensity as dsDNA is converted to ssDNA. We find that UvrD is capable of unwinding the 2.6-kb template (Figure 6A, B). As before we use linear fits to determine a rate for each trajectory (see methods). We find a broad distribution, with a median of 29.5 ± 28.3 bp·s^−1^ (median ± STD, see Figure 6C), consistent with values measured before in bulk and single-molecule studies (37,45,46). However, to our knowledge, this is the first study reporting unwinding of long (>100 bp) DNA substrates, despite its potential importance during biological processes, such as methyl-directed mismatch repair, which can require more than 1000 bp to be unwound (47).

## DISCUSSION

We report a highly generalisable and high-throughput single-molecule assay with fully automated data analysis to study DNA-based enzymatic processes. This assay allows the extraction of features and kinetics otherwise hidden in the noise of single-molecule measurements. To demonstrate the strengths of this assay, we characterised DNA degradation, synthesis, and unwinding. Furthermore, we observe removal of a DNA-bound neutravidin by the UvrD helicase. The characterisation of neutravidin or streptavidin removal from DNA by helicases has previously been shown to be a good model for disruption of general nucleoprotein complexes by helicases, (9,48), this new activity might therefore have physiological relevance.

Reproducibility of fluorescence microscopy methods was previously identified as a major issue (49). Quantitative fluorescence microscopy is inherently difficult to reproduce, due to the large number of factors involved. Fluorescence intensity varies dependent on the specific imaging apparatus, including the used light sources, as well as lenses and objectives and precise alignment thereof. Our assay produces data, in which mechanistic features are directly visible. This aspect allows for internal normalisation of fluorescence intensity and therefore circumvents this problem. Another factor of uncertainty in microscopy data is human bias during image analysis. We developed highly automated image analysis software for our assay, to minimise this problem.

Our method and analysis pipeline should be broadly applicable to measure the activity of any enzyme that converts dsDNA to ssDNA or *vice versa*. Furthermore, due to its high-throughput nature, the method has potential to be implemented in evolution or drug-screening studies.

## DATA AVAILABILITY

All data presented (DOI: 10.5281/zenodo.7042235), home-built ImageJ plugins (DOI: 10.5281/zenodo.7047425) and python scripts (DOI: 10.5281/zenodo.7047432) are available at Zenodo.org.

## Supplementary Material

gkac949_Supplemental_File
