# Supplementary material for: Rapid single-molecule characterisation of enzymes involved in nucleic-acid metabolism
Source: Nucleic Acids Res. 2022 Nov 2;51(1):e5. doi: 10.1093/nar/gkac949 (PMC9841422; doi:10.1093/nar/gkac949)
Supplement: gkac949_Supplemental_File [file gkac949_supplemental_file.docx]

**Rapid single-molecule characterisation of enzymes involved in nucleic-acid metabolism**

**Supplementary information**

*
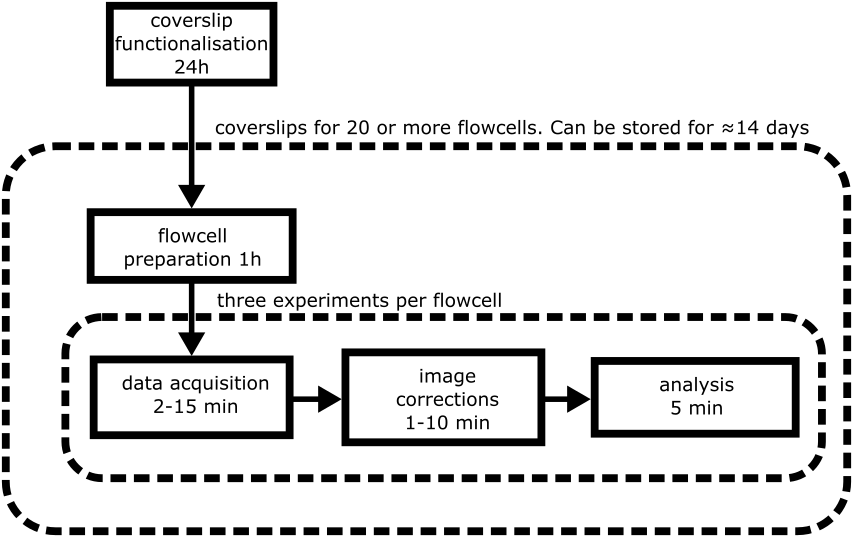
*

**Supplementary Fig. 1: Time-consumption of the described method:** Coverslip functionalisation: detailed description by Geertsema et al. (1). Flow cell preparation as described in methods or in greater detail by Geertsema et al. (1). However, the use of PDMS flow cells and syringe pumps is not a prerequisite for this assay. Simple flow cells can be manufactured using for example double-sided tape, see for example Brewer and Bianco (2) for more detailed descriptions. Data acquisition: largely governed by the turn-over rate of the studied enzyme. Image corrections: corrections as described in supplementary figures 3 and 4. The computational time is generally short, but can increase if large amounts of data are acquired. Analysis: Data analysis as described in methods, by using python scripts (see <https://doi.org/10.5281/zenodo.7047432> for source code)


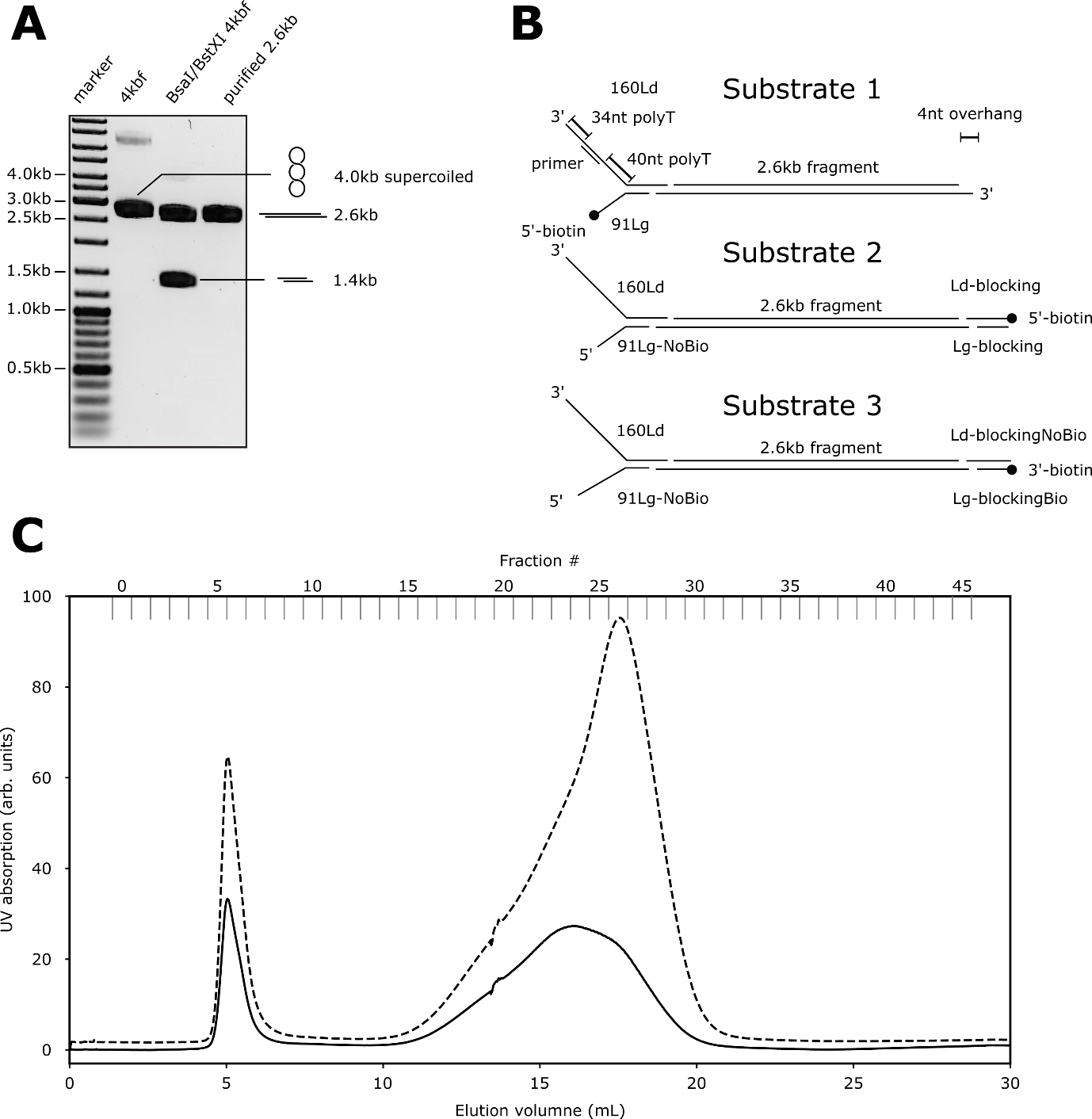


**Supplementary Fig. 2:** **Construction of 2.6kb DNA. (A)** 1% agarose gel, staining with 0.5 ug/mL EtBr. Lane 2 shows the supercoiled 4kbf plasmid, Lane 3 the digested plasmid including the 2.6kb fragment. The 2.6kb fragment was excised from a separate gel and is shown in lane 4. After ligation of blocking and fork oligonucleotides (see (**B)** and supplementary table 1) the final template was purified on a Sepharose 4B column. **(C)** Size-exclusion chromatogram showing UV280 absorption (solid line) and UV260 absorption (dashed line). The first peak corresponds to the 2.6kb DNA, the second peak consists of excess oligonucleotides and T4 ligase.

**
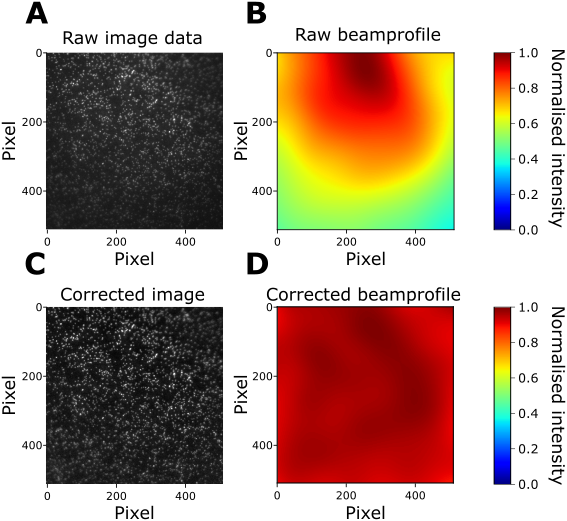
**

**Supplementary Fig. 3:** **Beam profile correction:** Our assay relies on reproducible and quantifiable fluorescence intensity measurement. However, uneven illumination of samples can lead to differences in fluorescence intensity within single images. **(A)** shows raw microscopic image data. Note that spots near the edges are considerably dimmer than spots close to the middle. **(B)** Applying a gaussian blur to the image evens out local intensity differences and yields the beam profile, here shown as a heatmap. **(C)** The raw image is corrected by dividing every pixel value by the beam profile. **(D)** The quality of the correction can be assessed by applying a gaussian blur once again. Comparing this beam profile to the raw beam profile (see (B)) one can see that large scale intensity fluctuations are now largely eliminated. To assure reusability of our method we implemented this correction in the form of an ImageJ plugin. We utilize the imglib2 java library (3) to achieve generic processing of single images, movies or multi-colour movies alike. See https://doi.org/10.5281/zenodo.7047425

for source code and download.


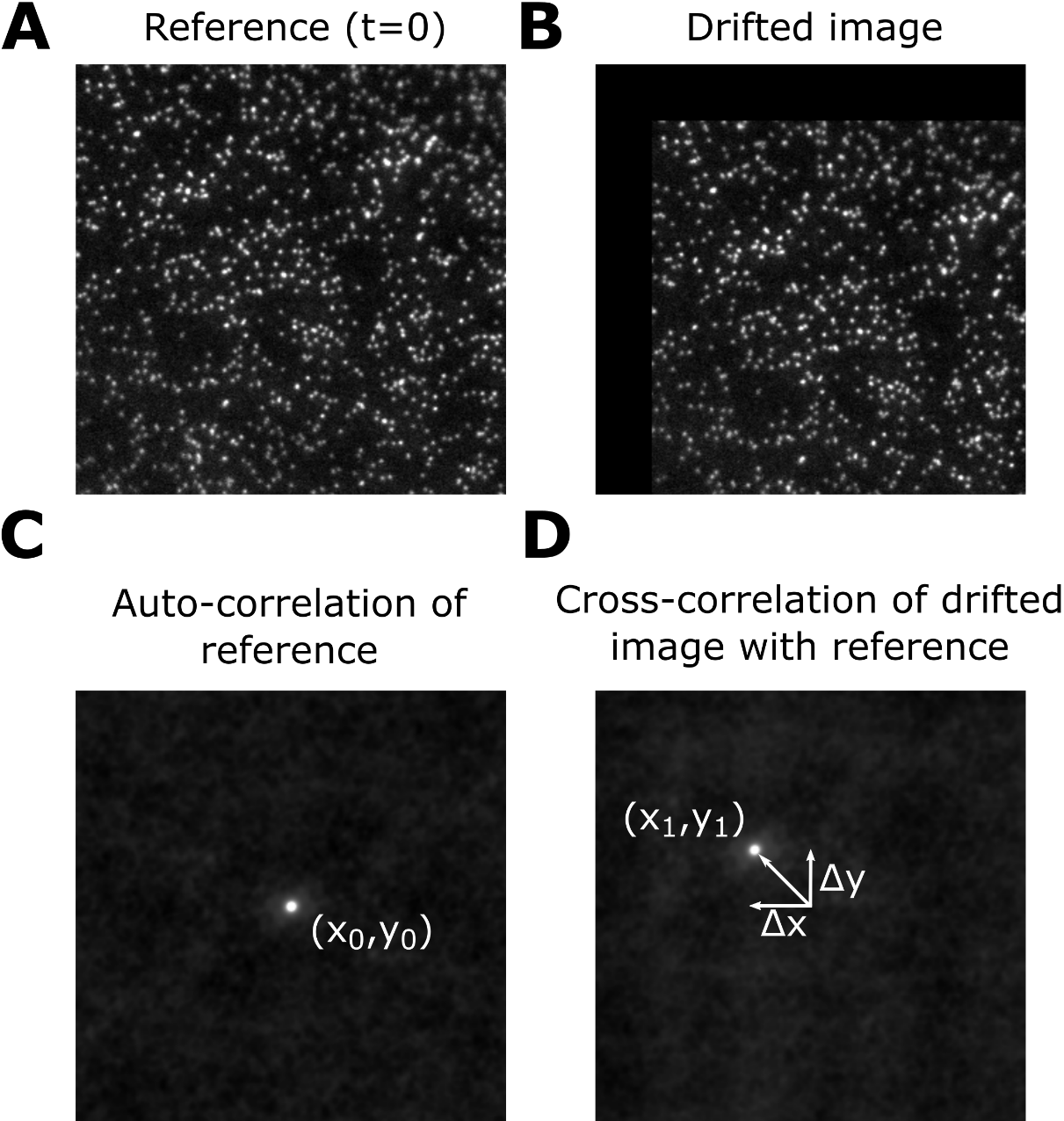


**Supplementary Fig. 4: Drift correction.** To correct for mechanical drift of the imaged sample we implement a template based correction algorithm. Briefly, the first frame of a movie **(A)** is treated as the reference. **(B)** shows the reference shifted in x and y direction to simulate drift. To find the total shift the cross-correlation **(D)** of the image with the reference is compared to the auto-correlation of the reference **(C)**. The shift-vector (Δx,Δy) is given by the change of position of the maximum value of the correlation images. For fast image-processing correlations are calculated in the Fourier-domain. We utilize the imglib2 java library (3) to achieve generic processing of single images, movies or multi-colour movies alike. See See https://doi.org/10.5281/zenodo.7047425 for source code and download.


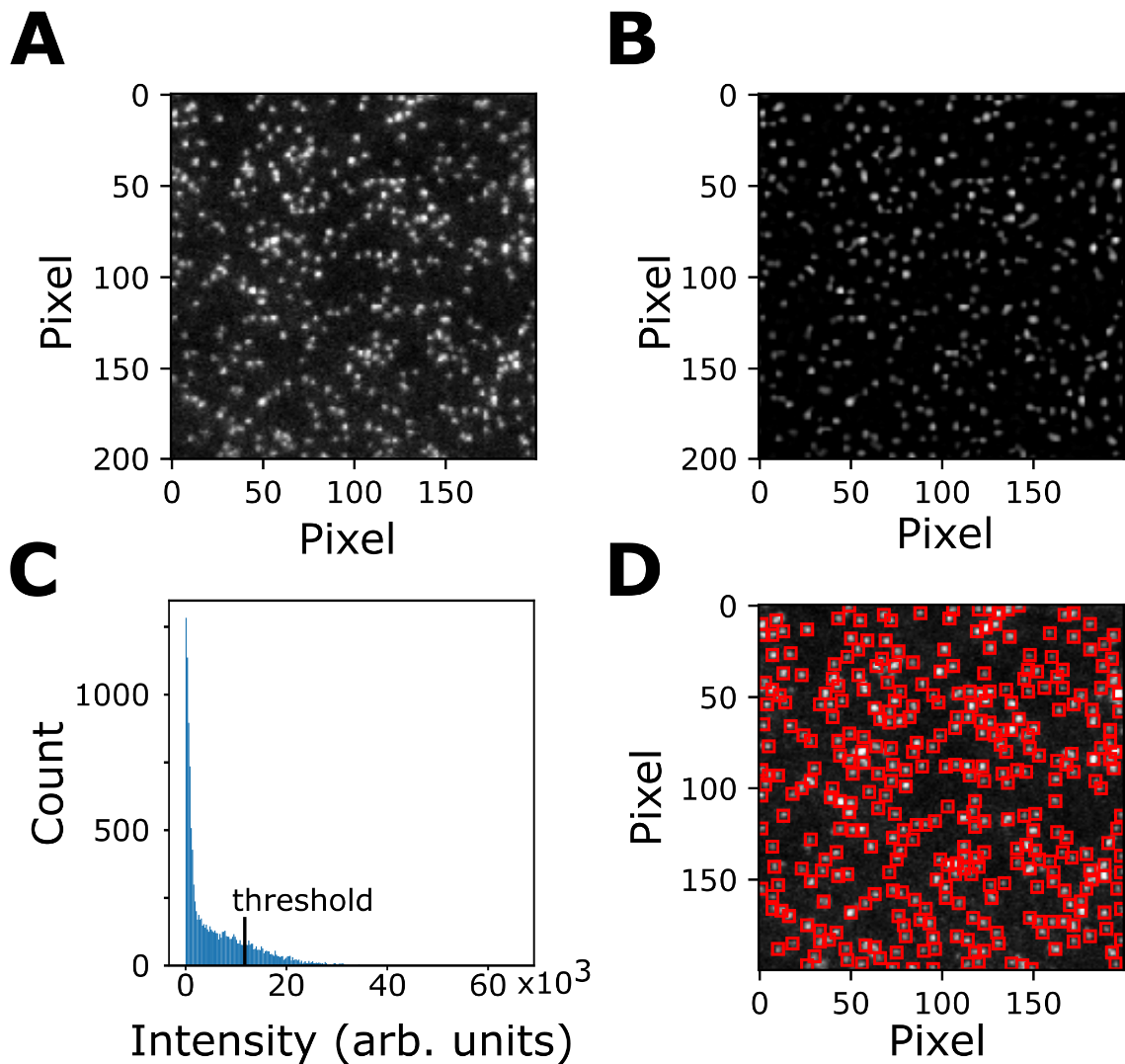


**Supplementary Fig. 5: Detection of events.** As descirbed in methods section, we use a thresholding approach to detect the signal of single DNA molecules. **(A)** shows a 200x200 pixel region from supplementary Fig 6C. Before threshold analysis a discoidal filter is applied. **(B)** Filtered image. The discoidal filter is a moving window filter. Every pixel’s grey value is substituted by the mean of all pixels within an inner radius r_in_. Subsequently the mean grey value of all pixels within a second outer region with radius r_out_ is subtracted. For the shown image r_in_ =1 and r_out_=3. This leads to greatly reduces background intensity. **(C)** histogram of grey values in **(B).** To detect peaks a threshold is set to be twice the standard deviation of the distribution of grey values, as indicated by the black bar. **(D)** detected peaks. Every pixel with a grey value above the threshold is defined as one peak. If multiple pixel coincide within a minimum distance of 6 pixels only the one with the higher grey value is counted as peak.

**
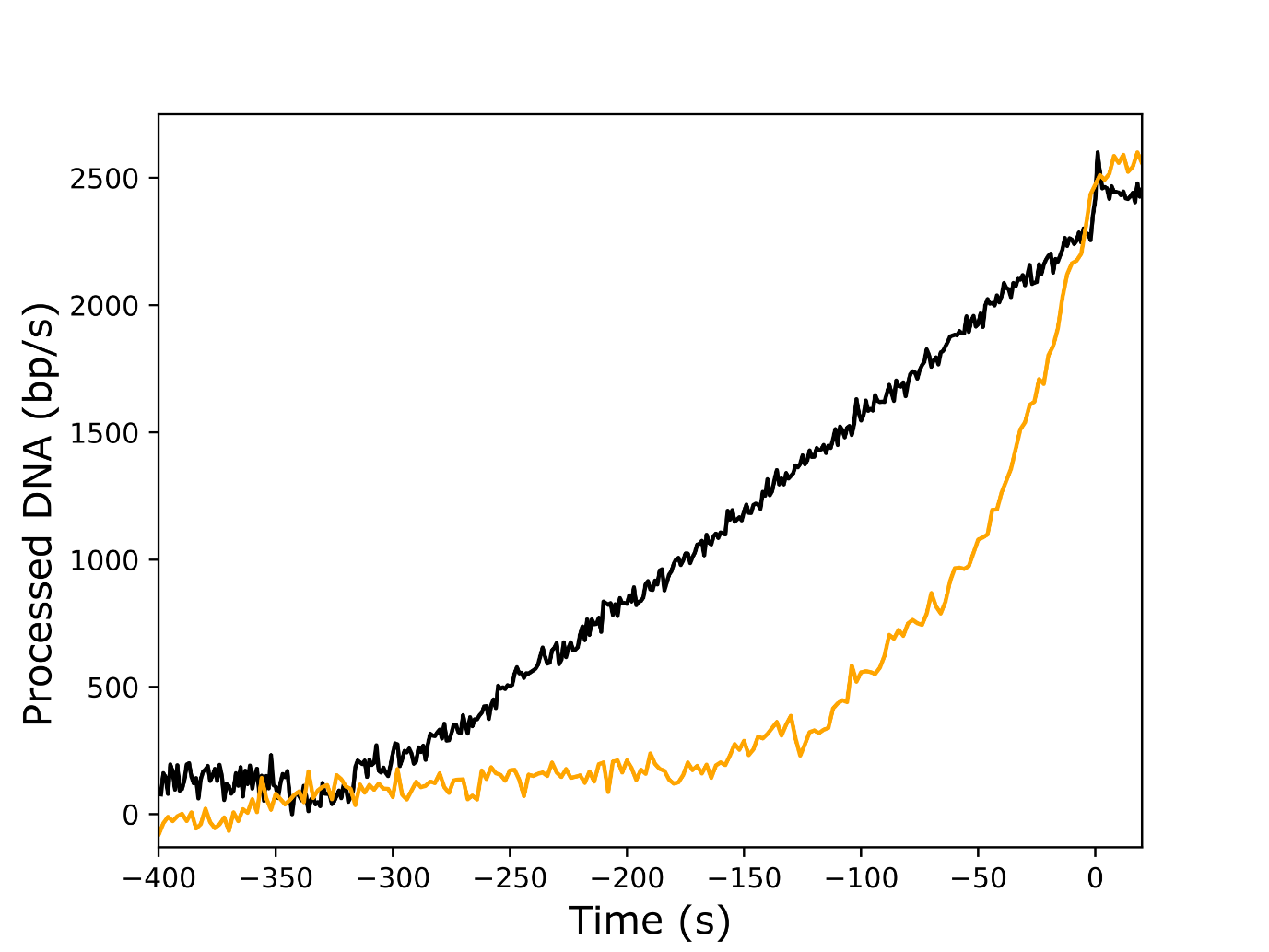
**

**Supplementary Fig. 6:** **Post-synchronised average trajectories.** To prove that the non-linear behaviour in Phi29 DNAp averages is not due to synchronisation, we synchronised λ exo trajectories, determining the end point of individual trajectories using a piecewise linear fit (see methods). The graph shows the inverted synchronised mean of 666 λ exo trajectories (black) to provide easy comparison to the synchronised mean of 127 Phi29 DNAp trajectories (orange).


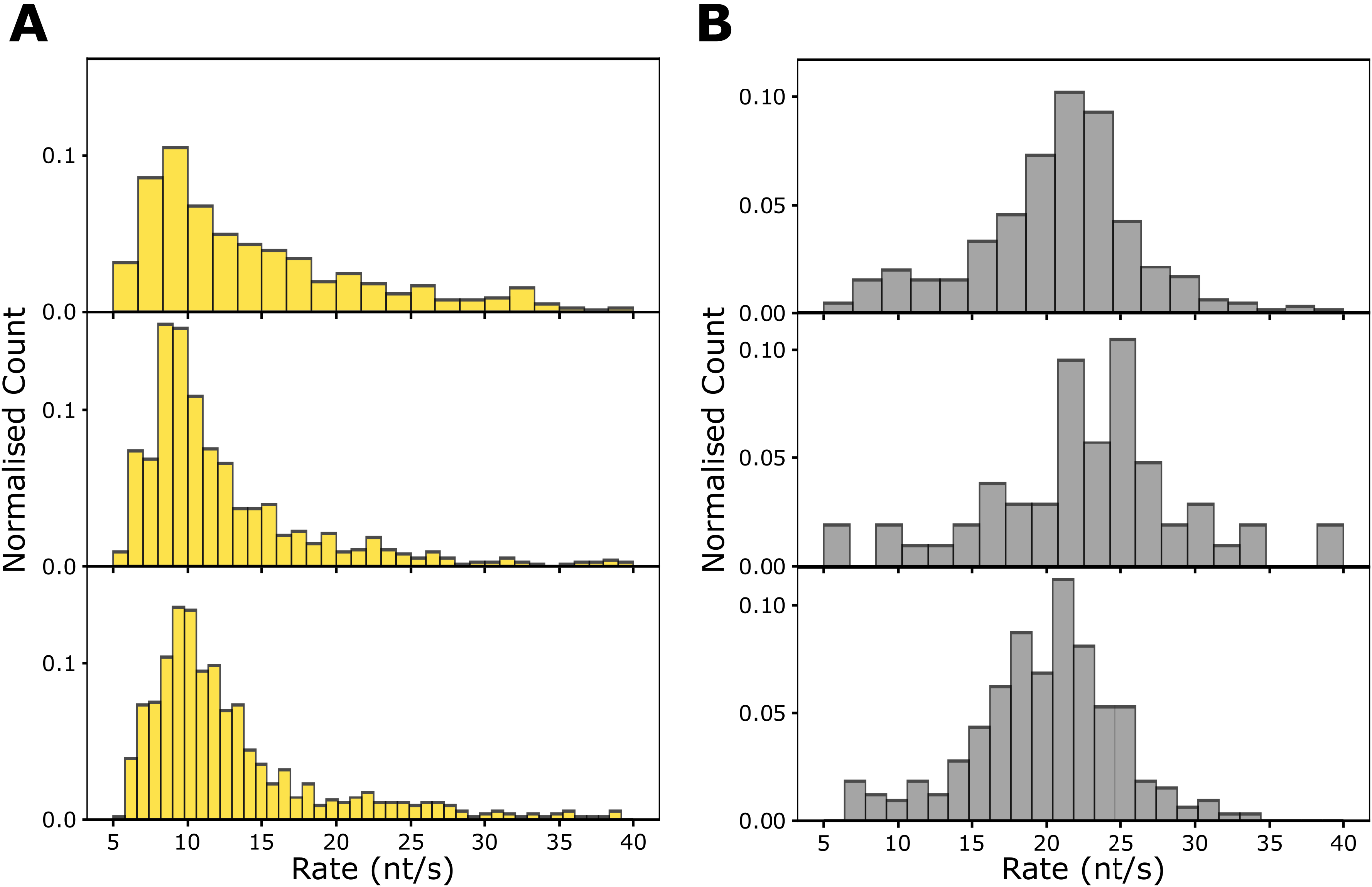


**Supplementary Fig 7: Datasets from individual experiments with λ exonuclease: (A)** rate distribution measured in three independent experiments at 25°C. From top to bottom: number of molecules n=469, mean±STD: 14.34±7.33 nt/s; n=764, mean±STD: 12.39±5.95 nt/s; n= 704, mean±STD: 13.01±6.22 nt/s **(B)** rate distribution measured in three independent experiments at 35 °C. From top to bottom: number of molecules n=339, mean±STD: 20.55±5.67 nt/s; n=62, mean±STD: 21.90±7.6 nt/s; n=231, mean±STD: 19.75±5.08 nt/s.

**
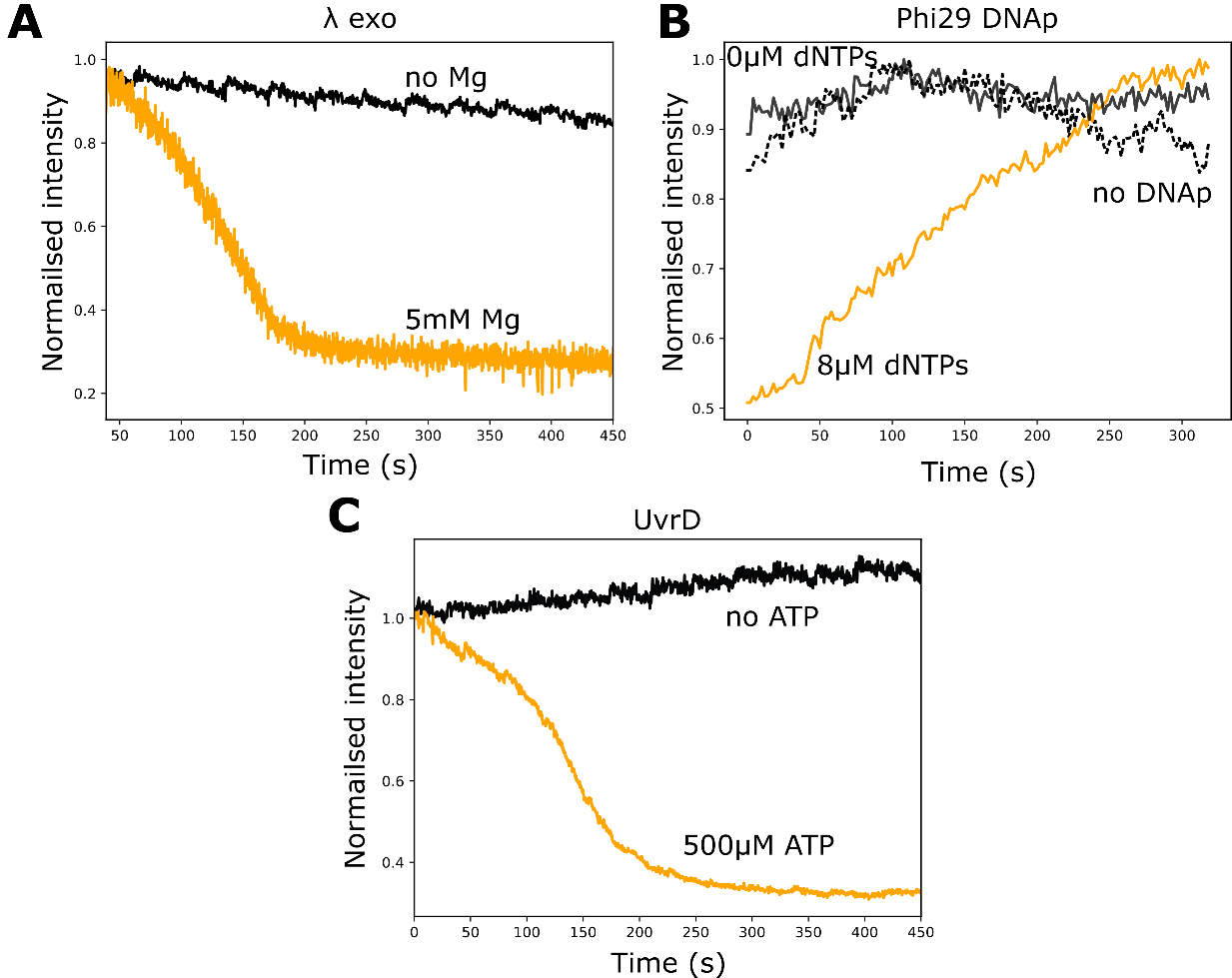
 Supplementary Fig. 8: Control reactions. (A)** To confirm the observed signal is due to λ exo activity we imaged in absence of Mg but otherwise identical conditions as stated in methods. This control also shows that photo-bleaching does not affect our quantification of dsDNA, since S.O. signal remains approximately constant over whole observed time scale in the absence of enzymatic activity. The shown trajectories are non-synchronised averages, orange line: no Mg, black line: with Mg. **(B)** non-synchronised averages of Phi29 DNAp trajectories (see methods) with 8 μM dNTPs (orange), 0 μM dNTPs (solid black line) and 8 μM dNTPs but in absence of Phi29 DNAp (dashed black line). The black lines also prove that our observed RPA kinetics are not influenced by photo-bleaching. They remain approximately constant in the absence of enzymatic activity. **(C)** UvrD on substrate 3 (see methods) in presence of 500 μM ATP (orange) and in absence of ATP (black), with a buffer flow of 10 μL/min**.**


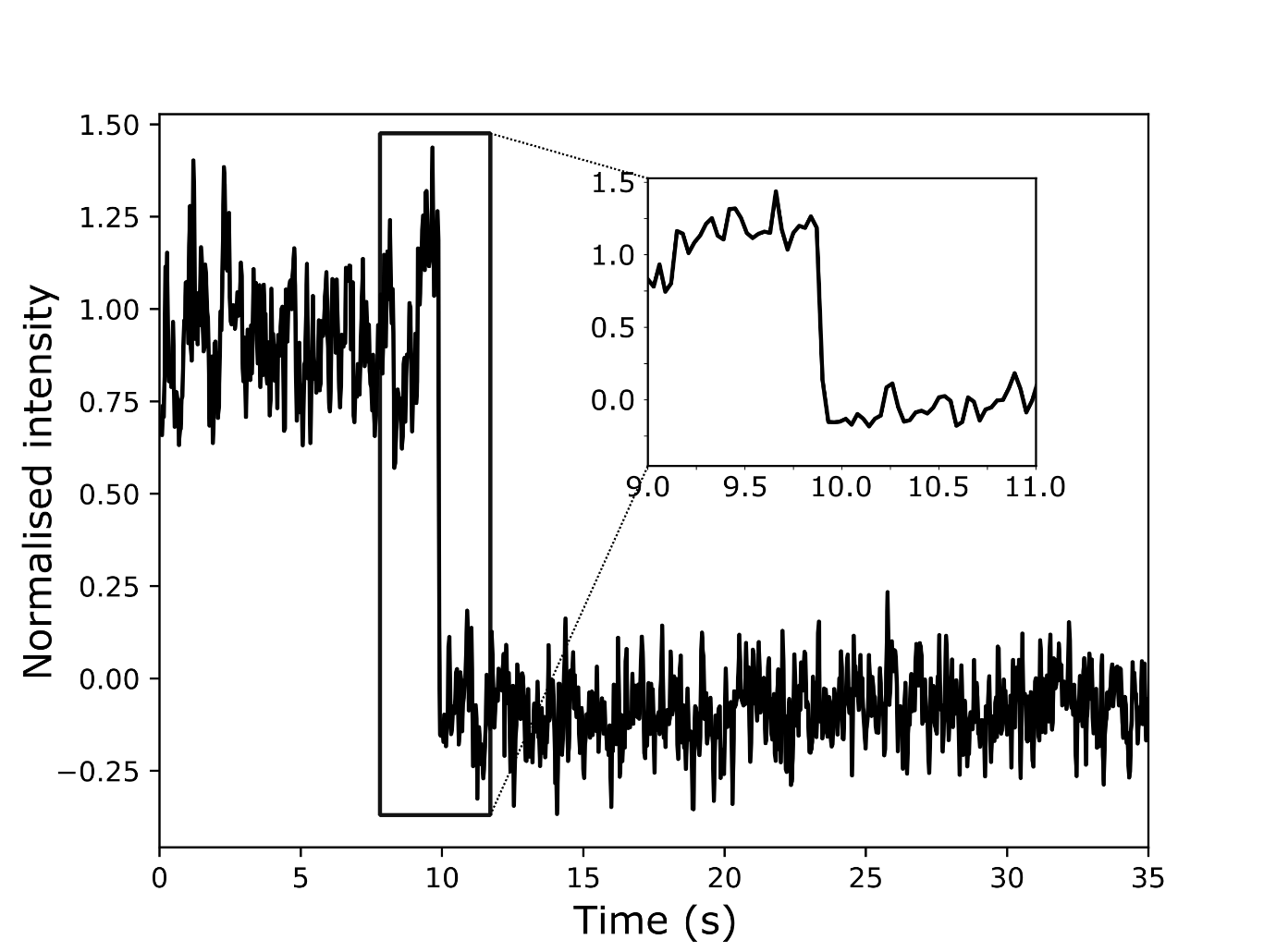


**Supplementary Fig. 9: High-Speed imaging of UvrD dependent dissociation of substrate 1.** Substrate 1, stained by S.O. was imaged in presence of 100 nM UvrD and 10 μM ATP with an exposure time of 30 ms and a laser intensity of 59 mW∙cm^2^. The laser intensity drops within one frame, i.e. 30 ms.

**
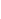
**


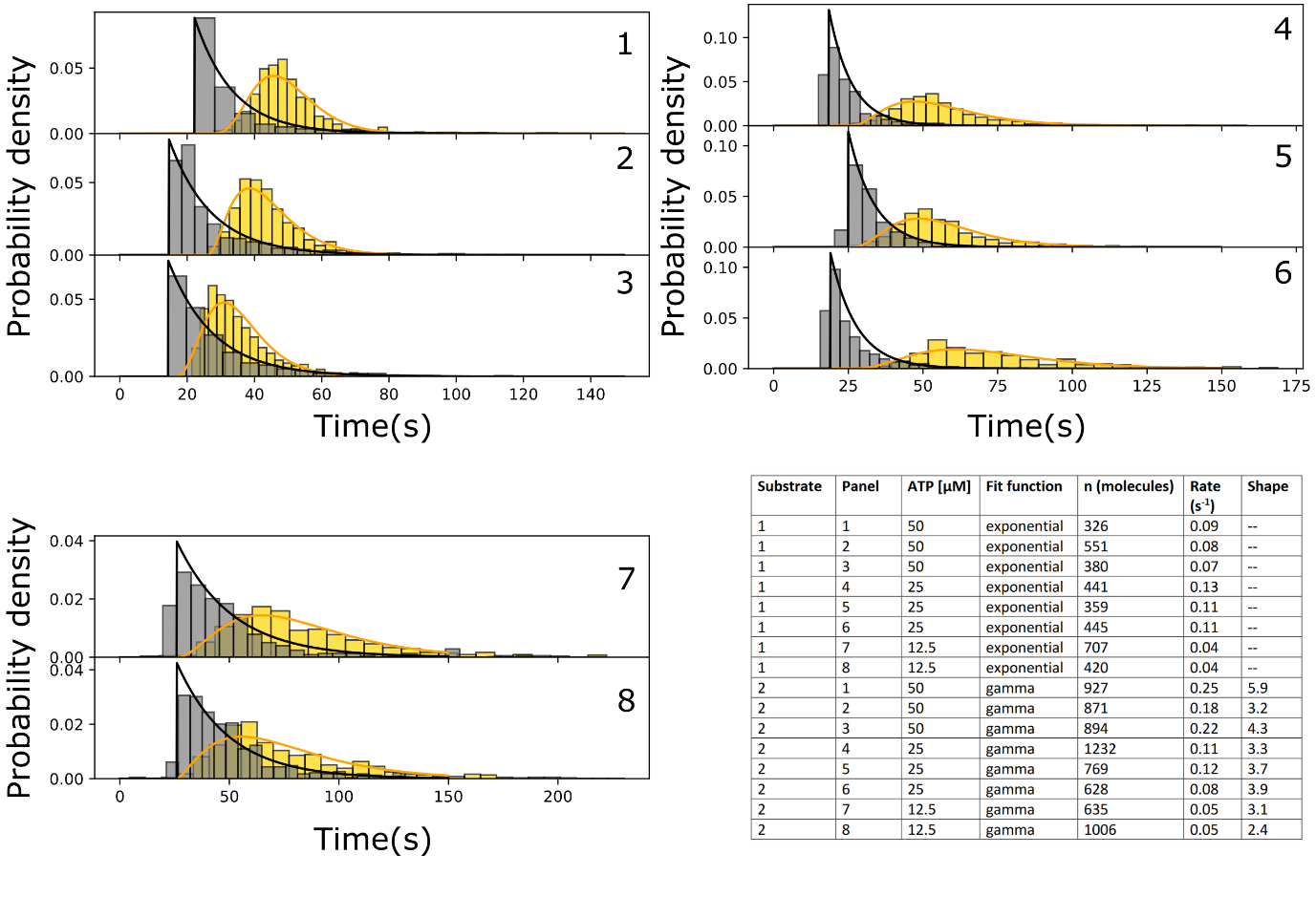


**Supplementary Fig 10:** **Datasets from individual experiments with UvrD on Substrates 1 and 2 (see methods)** All histograms are from physically independent experiments. Datasets on Substrate 1 (grey) are fitted to exponential distributions (black lines, see methods), datasets on Substrate 2 (yellow) are fitted to gamma distributions (orange lines, see methods). All panels containing histograms are numerically numbered, the table shows the corresponding results of all individual fits and the number of molecules corresponding to the histograms.

***Supplementary Table 1: Sequences of oligonucleotides used in this study.*** All oligonucleotides were purchased from IDT. /Bio/ refers to a biotin, covalently attached through a C6 spacer either to the 3**′** or the 5**′** end. /Phos/ refers to a phosphorylated 5**′**end.

| **Name (DNA)** | **Sequence (5′—3′)** |
| --- | --- |
| BlockingLdNoBio | AGT CGC AGC TAT AGG TGG CAT TTC AG |
| BlockingLd | /Bio/AGT CGC AGC TAT AGG TGG CAT TTC AG |
| BlockingLg | /Phos/CTG AAA TGC CAC CTA TAG CTG CGA CTC ATG |
| BlockingLgBio | /Phos/CTG AAA TGC CAC CTA TAG CTG CGA CTC ATG/Bio/ |
| 160Ld | /Phos/ACC GAT GTG GTA GGA AGT GAG AAT TGG AGA GTG TGT TTT TTT TTT TTT TTT TTT TTT TTT TTT TTT TTT TTT TTT GAG GAA AGA ATG TTG GTG AGG GTT GGG AAG TGG AAG GAT GGG CTC GAG AGG TTT TTT TTT TTT TTT TTT TTT TTT TTT TTT T*T*T*T |
| 91Lg | TTT TTT TTT TTT TTT TTT TTT TTT TTT TTT TTT TTT TTT TTT TTT TTT TTT TTT TTT TTT CAC ACT CTC CAA TTC TCA CTT CCT ACC ACA T |
| Fork Primer | CCT CTC GAG CCC ATC CTT CCA CTT CCC AAC CCT CAC C |

***Supplementary Table 2: Complete oligonucleotide sequence of 4kbf.***

| ACCGCGAG ACCCACGC TCACCGGC TCCAGATT TATCAGCA ATAAACCA GCCAGCCG GAAGGGCC GAGCGCAG AAGTGGTC CTGCAACT TTATCCGC CTCCATCC AGTCTATT AATTGTTG CCGGGAAG CTAGAGTA AGTAGTTC GCCAGTTA ATAGTTTG CGCAACGT TGTTGCCA TTGCTACA GGCATCGT GGTGTCAC GCTCGTCG TTTGGTAT GGCTTCAT TCAGCTCC GGTTCCCA ACGATCAA GGCGAGTT ACATGATC CCCCATGT TGTGCAAA AAAGCGGT TAGCTCCT TCGGTCCT CCGATCGT TGTCAGAA GTAAGTTG GCCGCAGT GTTATCAC TCATGGTT ATGGCAGC ACTGCATA ATTCTCTT ACTGTCAT GCCATCCG TAAGATGC TTTTCTGT GACTGGTG AGTACTCA ACCAAGTC ATTCTGAG AATAGTGT ATGCGGCG ACCGAGTT GCTCTTGC CCGGCGTC AATACGGG ATAATACC GCGCCACA TAGCAGAA CTTTAAAA GTGCTCAT CATTGGAA AACGTTCT TCGGGGCG AAAACTCT CAAGGATC TTACCGCT GTTGAGAT CCAGTTCG ATGTAACC CACTCGTG CACCCAAC TGATCTTC AGCATCTT TTACTTTC ACCAGCGT TTCTGGGT GAGCAAAA ACAGGAAG GCAAAATG CCGCAAAA AAGGGAAT AAGGGCGA CACGGAAA TGTTGAAT ACTCATAC TCTTCCTT TTTCAATA TTATTGAA GCATTTAT CAGGGTTA TTGTCTCA TGAGCGGA TACATATT TGAATGTA TTTAGAAA AATAAACA AATAGGGG TTCCGCGC ACATTTCC CCGAAAAG TGCCACCT GACGTCTA AGAAACCA TTATTATC ATGACATT AACCTATA AAAATAGG CGTATCAC GAGGCCCT TTCGTCTC GCGCGTTT CGGTGATG ACGGTGAA AACCTCTG ACACATGC AGCTCCCG GAGACGGT CACAGCTT GTCTGTAA GCGGATGC CGGGAGCA GACAAGCC CGTCAGGG CGCGTCAG CGGGTGTT GGCGGGTG TCGGGGCT GGCTTAAC TATGCGGC ATCAGAGC AGATTGTA CTGAGAGT GCACCATA TGCGGTGT GAAATACC GCACAGAT GCGTAAGG AGAAAATA CCGCATCA GGCGCCAT TCGCCATT CAGGCTGC GCAACTGT TGGGAAGG GCGATCGG TGCGGGCC TCTTCGCT ATTACGCC AGCTGGCG AAAGGGGG ATGTGCTG CAAGGCGA TTAAGTTG GGTAACGC CAGGGTTT TCCCAGTC ACGACGTT GTAAAACG ACGGCCAG TGAATTCG AGCTCGGA TGTTTTGG CTCTGGTC AATGATTA CGGCATTG ATATCGTC CAACTGCA TGGAGATG AGTCGTGG CAAGAATA CCAAGAGT TCCTCGGT TTGCCAGT TATTAAAA GACTCGTA TTTCCAAA AGACTGCA ACATACTA CTCAGTGC AGCTTCAC AGAAACCT CATTCGTT TATTCCCT TGTTTGAT TCAGAAGC AGGTGGGA CAGGTGAA CTTTTGGA TTGGAACT CGATTTCT GACTGGGT TGGAAGGC AAGAGAGC CCCGAAAG CTTACATT TTATGTTA GCTGGTGG ACTGACGC CAGAAAAT GTTGGTGA TGCGCTTA GATTAAAT GGCGTTAT TGGTGTTG ATGTAAGC GGAGGTGT GGAGACAA ATGGTGTA AAAGACTC TAACAAAA TAGCAAAT TTCGTCAA AAATGCTA AGAAATAG GTTATTAC TGAGTAGT ATTTATTT AAGTATTG TTTGTGCA CTTGCCTG CAGGCCTT TTGAAAAG CAAGCATA AAAGATCT AAACATAA AATCTGTA AAATAACA AGATGTAA AGATAATG CTAAATCA TTTGGCTT TTTGATTG ATTGTACA GGAAAATA TACATCGC AGGGGGTT GACTTTTA CCATTTCA CCGCAATG GAATCAAA CTTGTTGA AGAGAATG TTCACAGG CGCATACG CTACAATG ACCCGATT CTTGCTAG CCTTTTCT CGGTCTTG CAAACAAC CGCCGGCA GCTTAGTA TATAAATA CACATGTA CATACCTC TCTCCGTA TCCTCGTA ATCATTTT CTTGTATT TATCGTCT TTTCGCTG TAAAAACT TTATCACA CTTATCTC AAATACAC TTATTAAC CGCTTTTA CTATTATC TTCTACGC TGACAGTA ATATCAAA CAGTGACA CATATTAA ACACAGTG GTTTCTTT GCATAAAC ACCATCAG CCTCAAGT CGTCAAGT AAAGATTT CGTGTTCA TGCAGATA GATAACAA TCTATATG TTGATAAT TAGCGTTG CCTCATCA ATGCGAGA TCCGTTTA ACCGGACC CTAGTGCA CTTACCCC ACGTTCGG TCCACTGT GTGCCGAA CATGCTCC TTCACTAT TTTAACAT GTGGAATT CTTGAAAG AATGAGTT CAGTGGTG CTGACACG ATATTTTA TTCATACA ACATTGAT TTCACCAA TTATAATA CCGCGCAA TACTGTAT GAGCATAC AGTGGATC CGAGTCAT TCCTGCAG CGAGTCCA TGGGAGTC AAATAGAC AACGATTT GAATTCGC TCTTCCGC TCGCGGCC GCTAAGAC TCGAGTAG ATGACTAC GAGGTACC CGGGGATC CTCTAGAG TCGACCTG CAGGCATG CAAGCTTG GCGTAATC ATGGTCAT AGCTGTTT CCTGTGTG AAATTGTT ATCCGCTC ACAATTCC ACACAACA TACGAGCC GGAAGCAT AAAGTGTA AAGCCTGG GGTGCCTA ATGAGTGA GCTAACTC ACATTAAT TGCGTTGC GCTCACTG CCCGCTTT CCAGTCGG GAAACCTG TCGTGCCA GCTGCATT AATGAATC GGCCAACG CGCGGGGA GAGGCGGT TTGCGTAT TGGGCGCT CTTCCGCT TCCTCGCT CACTGACT CGCTGCGC TCGGTCGT TCGGCTGC GGCGAGCG GTATCAGC TCACTCAA AGGCGGTA ATACGGTT ATCCACAG AATCAGGG GATAACGC AGGAAAGA ACATGTGA GCAAAAGG CCAGCAAA AGGCCAGG AACCGTAA AAAGGCCG CGTTGCTG GCGTTTTT CCATAGGC TCCGCCCC CCTGACGA GCATCACA AAAATCGA CGCTCAAG TCAGAGGT GGCGAAAC CCGACAGG ACTATAAA GATACCAG GCGTTTCC CCCTGGAA GCTCCCTC GTGCGCTC TCCTGTTC CGACCCTG CCGCTTAC CGGATACC TGTCCGCC TTTCTCCC TTCGGGAA GCGTGGCG CTTTCTCA TAGCTCAC GCTGTAGG TATCTCAG TTCGGTGT AGGTCGTT CGCTCCAA GCTGGGCT GTGTGCAC GAACCCCC CGTTCAGC CCGACCGC TGCGCCTT ATCCGGTA ACTATCGT CTTGAGTC CAACCCGG TAAGACAC GACTTATC GCCACTGG CAGCAGCC ACTGGTAA CAGGATTA GCAGAGCG AGGTATGT AGGCGGTG CTACAGAG TTCTTGAA GTGGTGGC CTAACTAC GGCTACAC TAGAAGAA CAGTATTT GGTATCTG CGCTCTGC TGAAGCCA GTTACCTT CGGAAAAA GAGTTGGT AGCTCTTG ATCCGGCA AACAAACC ACCGCTGG TAGCGGTG GTTTTTTT GTTTGCAA GCAGCAGA TTACGCGC AGAAAAAA AGGATCTC AAGAAGAT CCTTTGAT CTTTTCTA CGGGGTCT GACGCTCA GTGGAACG AAAACTCA CGTTAAGG GATTTTGG TCATGAGA TTATCAAA AAGGATCT TCACCTAG ATCCTTTT AAATTAAA AATGAAGT TTTAAATC AATCTAAA GTATATAT GAGTAAAC TTGGTCTG ACAGTTAC CAATGCTT AATCAGTG AGGCACCT ATCTCAGC GATCTGTC TATTTCGT TCATCCAT AGTTGCCT GACTCCCC GTCGTGTA GATAACTA CGATACGG GAGGGCTT ACCATCTG GCCCCAGT GCTGCAAT GAT |
| --- |

**Supplementary References**

1. Geertsema,H.J., Duderstadt,K.E. and van Oijen,A.M. (2015) Single-Molecule Observation of Prokaryotic DNA Replication. *Methods Mol Biol*, **1300**, 219–238.

2. Brewer,L.R. and Bianco,P.R. (2008) Laminar flow cells for single-molecule studies of DNA-protein interactions. *Nat. Methods*, **5**, 517–525.

3. Pietzsch,T., Preibisch,S., Tomančák,P. and Saalfeld,S. (2012) ImgLib2—generic image processing in Java. *Bioinformatics*, **28**, 3009–3011.
